# Neural signal propagation atlas of *Caenorhabditis elegans*

**DOI:** 10.1038/s41586-023-06683-4

**Published:** 2023-11-01

**Authors:** Francesco Randi, Anuj K. Sharma, Sophie Dvali, Andrew M. Leifer

**Affiliations:** 1 Department of Physics, Princeton University, Princeton, NJ USA; 2 Princeton Neurosciences Institute, Princeton University, Princeton, NJ USA; 3 Present Address: Regeneron Pharmaceuticals, Tarrytown, NY USA

**Keywords:** Neural circuits, Optogenetics, Fluorescence imaging, Biophysical models, Biological physics

## Abstract

Establishing how neural function emerges from network properties is a fundamental problem in neuroscience^1^. Here, to better understand the relationship between the structure and the function of a nervous system, we systematically measure signal propagation in 23,433 pairs of neurons across the head of the nematode *Caenorhabditis elegans* by direct optogenetic activation and simultaneous whole-brain calcium imaging. We measure the sign (excitatory or inhibitory), strength, temporal properties and causal direction of signal propagation between these neurons to create a functional atlas. We find that signal propagation differs from model predictions that are based on anatomy. Using mutants, we show that extrasynaptic signalling not visible from anatomy contributes to this difference. We identify many instances of dense-core-vesicle-dependent signalling, including on timescales of less than a second, that evoke acute calcium transients—often where no direct wired connection exists but where relevant neuropeptides and receptors are expressed. We propose that, in such cases, extrasynaptically released neuropeptides serve a similar function to that of classical neurotransmitters. Finally, our measured signal propagation atlas better predicts the neural dynamics of spontaneous activity than do models based on anatomy. We conclude that both synaptic and extrasynaptic signalling drive neural dynamics on short timescales, and that measurements of evoked signal propagation are crucial for interpreting neural function.

## Abstract (web summary)

Measurements of signal propagation in more than 23,000 pairs of neurons from nematode worms show that predictions of neural function made on the basis of anatomy are often incorrect, in part owing to the effects of extrasynaptic signalling.

## Main

Brain connectivity mapping is motivated by the claim that “nothing defines the function of a neuron more faithfully than the nature of its inputs and outputs”^2^. This approach to revealing neural function drives large-scale efforts to generate connectomes—anatomical maps of the synaptic contacts of the brain—in a diverse set of organisms, ranging from mice^3^ to *Platynereis*^4^. The *C. elegans* connectome^1,5,6^ is the most mature of these efforts, and has been used to reveal circuit-level mechanisms of sensorimotor processing^7,8^, to constrain models of neural dynamics^9^ and to make predictions of neural function^10^.

Anatomy, however, omits key aspects of neurons’ inputs and outputs, or leaves them ambiguous: the strength and sign (excitatory or inhibitory) of a neural connection are not always evident from wiring or gene expression. Many mammalian neurons release both excitatory and inhibitory neurotransmitters, and functional measurements are thus required to disambiguate their connections^11^. For example, starburst amacrine cells release both GABA (γ-aminobutyric acid) and acetylcholine^12^; neurons in the dorsal raphe nucleus release both serotonin and glutamate^13^; and neurons in the ventral tegmental area release two or more of dopamine, GABA and glutamate^14^. The timescale of neural signalling is also ambiguous from anatomy. In addition, anatomy disregards changes to neural connections from plasticity or neuromodulation; for example, in the head compass circuit in *Drosophila*^15^ or in the crab stomatogastric ganglion^16^, respectively. Both mechanisms serve to strengthen or to select subsets of neural connections out of a menu of possible latent circuits. Finally, anatomy ignores neural signalling that occurs outside the synapse, as explored here. These ambiguities or omissions all pose challenges for revealing neural function from anatomy.

A more direct way to probe neural function is to measure signal propagation by perturbing neural activity and measuring the responses of other neurons. Measuring signal propagation captures the strength and sign of neural connections reflecting plasticity, neuromodulation and even extrasynaptic signalling. Moreover, direct measures of signal propagation allow us to define mathematical relations that describe how the activity of an upstream neuron drives activity in a downstream neuron, including its temporal response profile. Historically, this and related perturbative approaches have been called many names (Supplementary Information), but they all stand in contrast to correlative approaches that seek to infer neural function from activity correlations alone. Correlative approaches do not directly measure causality and are limited to finding relations among only those neurons that happen to be active. Perturbative approaches measure signal propagation directly, but previous efforts have been restricted to selected circuits or subregions of the brain, and have often achieved only cell-type and not single-cell resolution^17–22^.

Here we use neural activation to measure signal propagation between neurons throughout the head of *C. elegans* at single-cell resolution. We survey 23,433 pairs of neurons—the majority of the possible pairs in the head—to present a systematic atlas. We show that functional measurements better predict spontaneous activity than anatomy does, and that peptidergic extrasynaptic signalling contributes to neural dynamics by performing a functional role similar to that of a classical neurotransmitter.

## Population imaging and single-cell activation

**Fig. 1:**
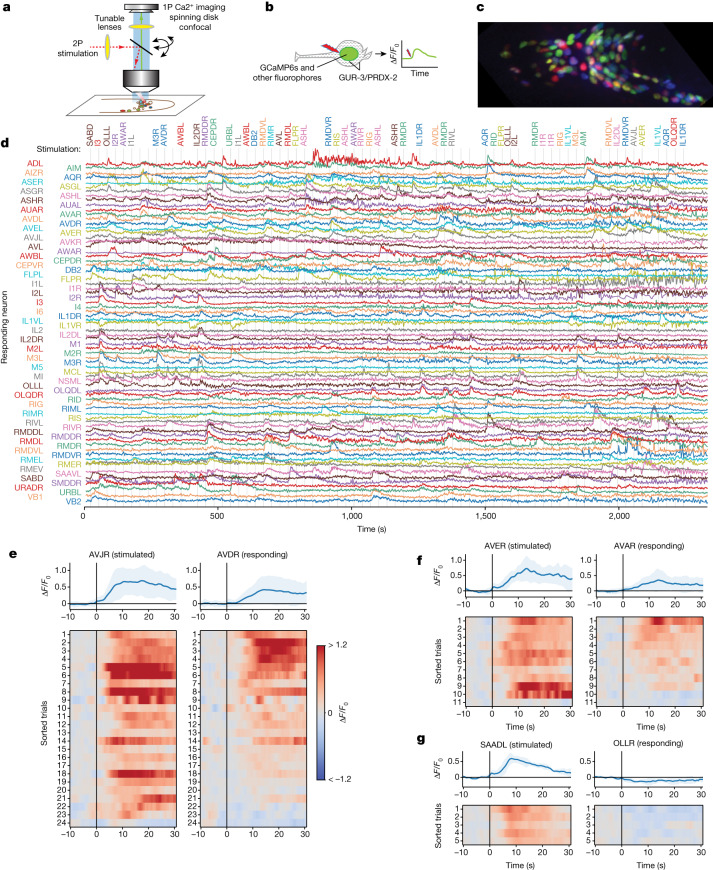
Measuring neural activation and network response. **a**,**b**, Schematics of the instrument (**a**) and the experiment (**b**). **c**, NeuroPAL fluorophores for neural identification. **d**, Whole-brain cell-resolved calcium activity (GCaMP6s fluorescence normalized by noise) during stimulation of individual neurons. A stimulation was delivered once every 30 s; grey lines indicate those instances when the stimuli were delivered on-target. The targeted neurons are listed at the top. **e**, Paired activity of AVJR and AVDR in response to AVJR stimulation, shown as relative change (Δ*F*/*F*_0_). Top, mean (blue) and s.d. (shading) across trials and animals. Bottom, simultaneously recorded paired activity for individual trials (sorted by mean AVDR activity). All trials are shown that elicited activity. **f**, Same as **e** for AVER stimulation and AVAR response. **g**, Same as **e** for SAADL stimulation and OLLR response.

To measure signal propagation, we activated each single neuron, one at a time, through two-photon stimulation, while simultaneously recording the calcium activity of the population at cellular resolution using spinning disk confocal microscopy (Fig. 1). We recorded activity from 113 wild-type (WT)-background animals, each for up to 40 min, while stimulating a mostly randomly selected sequence of neurons one by one every 30 s. We spatially restricted our two-photon activation in three dimensions to be the size of a typical *C. elegans* neuron, to minimize off-target activation of neighbouring neurons (Extended Data Fig. 2a,c–e,i,j and Supplementary Information). Animals were immobilized but awake, and pharyngeal pumping was visible during recordings. To overcome the challenges associated with spectral overlap between the actuator and the indicator, we used TWISP—a transgenic worm for interrogating signal propagation^23^, which expresses a purple-light actuator, GUR-3/PRDX-2 (refs. ^24,25^) and a nuclear-localized calcium indicator GCaMP6s (ref. ^26^) in each neuron (Fig. 1b and Extended Data Fig. 2b), along with fluorophores for neural identification from NeuroPAL (ref. ^27^) (Fig. 1c). Validation of the GUR-3/PRDX-2 system is discussed in the Supplementary Information (see also Extended Data Fig. 2h and Supplementary Video 1). A drug-inducible gene-expression system was used to avoid toxicity during development, resulting in animals that were viable but still significantly less active than WT animals^23^ (see Methods). A stimulus duration of 0.3 s or 0.5 s was chosen to evoke modest calcium responses (Extended Data Fig. 2f), similar in amplitude to those evoked naturally by odour stimuli^28^.

Many neurons exhibited calcium activity in response to the activation of one or more other neurons (Fig. 1d). A downstream neuron’s response to a stimulated neuron is evidence that a signal propagated from the stimulated neuron to the downstream neuron.

We highlight three examples from the motor circuit (Fig. 1e–g). Stimulation of the interneuron AVJR evoked activity in AVDR (Fig. 1e). AVJ had been predicted to coordinate locomotion after egg-laying by promoting forward movements^29^. The activity of AVD is associated with sensory-evoked (but not spontaneous) backward locomotion^7,8,30,31^, and AVD receives chemical and electrical synaptic input from AVJ^1,6^. Therefore, both wiring and our functional measurements suggest that AVJ has a role in coordinating backward locomotion, in addition to its previously described roles in egg-laying and forward locomotion.

Activation of the premotor interneuron AVER evoked activity transients in AVAR (Fig. 1f). Both AVA^31–35^ (Extended Data Fig. 2h) and AVE^31,36^ are implicated in backward movement. Their activities are correlated^31^, and AVE makes gap-junction and many chemical synaptic contacts with AVA^1,6^.

Activation of the turning-associated neuron SAADL^36^ inhibited the activity of the sensory neuron OLLR. SAAD had been predicted to inhibit OLL, on the basis of gene-expression measurements^37^. SAAD is cholinergic and it makes chemical synapses to OLL, which expresses an acetylcholine-gated chloride channel, LGC-47 (refs. ^6,38,39^). Other examples consistent with the literature are reported in Extended Data Table 1.

## Signal propagation map

**Fig. 2:**
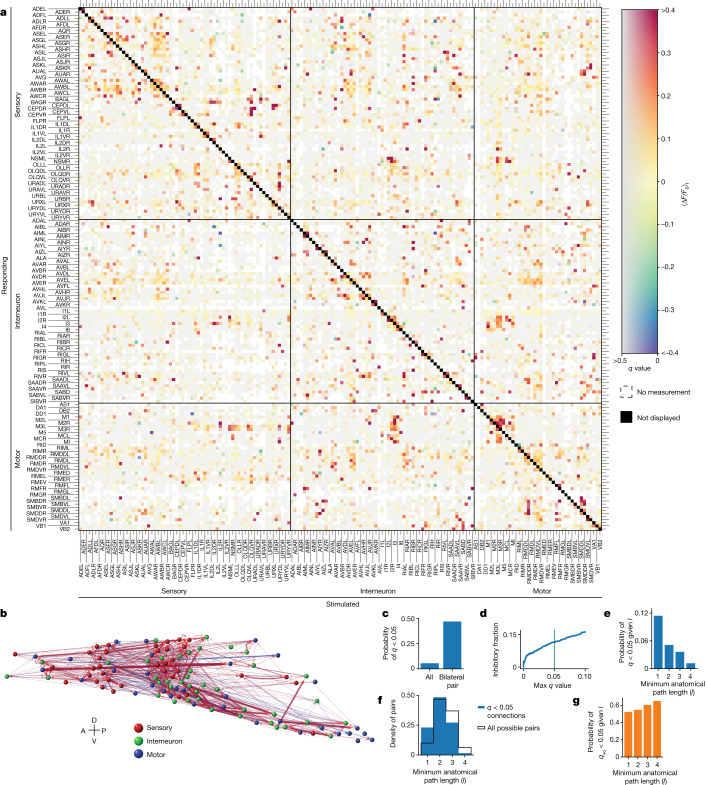
Signal propagation map of *C. elegans*. **a**, Mean post-stimulus neural activity ${\langle \Delta F/{F}_{0}\rangle }_{t}$ averaged across trials and individuals. The *q* values report the false discovery rate (more grey is less significant). White indicates no measurement. An autoresponse is required for inclusion and is not shown (black diagonal). *n* = 113 animals. Neurons that were recorded but never stimulated are shown in Extended Data Fig. 5. **b**, Corresponding network graph with neurons positioned anatomically (only *q* < 0.05 connections). Width and transparency indicate mean response amplitude (red, excitatory; blue, inhibitory). A, anterior; D, dorsal; P, posterior; V, ventral. **c**, A bilaterally symmetric pair is more likely to have a *q* < 0.05 functional connection than is a pair chosen at random. **d**, Fraction of connections that are inhibitory as a function of the *q*-value threshold. Green indicates *q* < 0.05. **e**, Probability of being functionally connected (*q* < 0.05) given minimum anatomical path length *l*. **f**, Distribution of *l* for functionally connected pairs (blue) compared to all possible pairs (black). **g**, Probability of being functionally non-connected (*q*_eq_ < 0.05) given *l*. Source Data

We generated a signal propagation map by aggregating downstream responses to stimulation from 113 *C. elegans* individuals (Fig. 2a). We report the mean calcium response in a 30-s time window ${\langle \Delta F/{F}_{0}\rangle }_{t}$ averaged across trials and animals (Extended Data Fig. 3a). We imaged activity in response to stimulation for 23,433 pairs of neurons (66% of all possible pairs in the head). Measured pairs were imaged at least once, and some as many as 59 times (Extended Data Figs. 3b and 4a). This includes activity from 186 of 188 neurons in the head, or 99% of all head neurons.

We developed a statistical framework, described in the Methods, to identify neuron pairs that can be deemed ‘functionally connected’ (*q* < 0.05; Extended Data Fig. 4b), ‘functionally non-connected’ (*q*_eq_ < 0.05; Extended Data Fig. 5b) or for which we lack the confidence to make either determination. The statistical framework is conservative and requires consistent and reliable responses (or non-responses) compared to an empirical null distribution, considering effect size, sample size and multiple-hypothesis testing^40^ to make either determination. Many neuron pairs fail to pass either statistical test, even though they often contain neural activity that, when observed in isolation, could easily be classified as a response (for example, AVJR→ASGR in Extended Data Fig. 4c).

Our signal propagation map comprises the response amplitude and its associated *q* value (Fig. 2a and Extended Data Fig. 5a) and can be browsed online (https://funconn.princeton.edu) through software built on the NemaNode platform^6^. A total of 1,310 of the 23,433 measured neuron pairs, or 6%, pass our stringent criteria to be deemed functionally connected at *q* < 0.05 (Fig. 2c). Neuron pairs that are deemed functionally non-connected are reported in Extended Data Fig. 5b. Note that, in all cases, functional connections refer to ‘effective connections’ because they represent the propagation of signals over all paths in the network between the stimulated and the responding neuron, not just the direct (monosynaptic) connections between them.

*C. elegans* neuron subtypes typically consist of two bilaterally symmetric neurons, often connected by gap junctions, that have similar wiring^1^ and gene expression^38^, and correlated activity^41^. As expected, bilaterally symmetric neurons are (eight times) more likely to be functionally connected than are pairs of neurons chosen at random (Fig. 2c).

The balance of excitation and inhibition is important for a network’s stability^42,43^ but has not to our knowledge been previously measured in the worm. Our measurements indicate that 11% of *q* < 0.05 functional connections are inhibitory (Fig. 2d), comparable to previous estimates of around 20% of synaptic contacts in *C. elegans*^37^ or around 20% of cells in the mammalian cortex^44^. Our estimate is likely to be a lower bound, because we assume that we only observe inhibition in neurons that already have tonic activity.

As expected from anatomy, neuron pairs that had direct (monosynaptic) wired connections were more likely to be functionally connected than were neurons with only indirect or multi-hop anatomical connections. Similarly, the likelihood of functional connections decreased as the minimal path length through the anatomical network increased (Fig. 2e). Conversely, neurons that had large minimal path lengths through the anatomical network were more likely to be functionally non-connected than were neurons that had a single-hop minimal path length (Fig. 2g). We investigated how far responses to neural stimulation penetrate into the anatomical network. Functionally connected (*q* < 0.05) neurons were on average connected by a minimal anatomical path length of 2.1 hops (Fig. 2f), suggesting that neural signals often propagate multiple hops through the anatomical network or that neurons are also signalling through non-wired means.

Most neuron pairs exhibited variability across trials and animals: downstream neurons responded to some instances of upstream stimulations but not others (Extended Data Fig. 6a); and the response’s amplitude, temporal shape and even sign also varied (Extended Data Fig. 6b–e). Some variability in the downstream response can be attributed to variability in the upstream neuron’s response to its own stimulation, called its autoresponse. To study the variability of signal propagation excluding variability from the autoresponse, we calculated a kernel for each stimulation that evoked a downstream response. The kernel gives the activity of the downstream neuron when convolved with the activity of the upstream neuron. The kernel describes how the signal is transformed from upstream to downstream neuron for that stimulus event, including the timescales of the signal transfer (Extended Data Fig. 6b,c). We characterized the variability of each functional connection by comparing how these kernels transform a standard stimulus (Extended Data Fig. 6e). Kernels for many neuron pairs varied across trials and animals, presumably because of state- and history-dependent effects^45^, including from neuromodulation^16,46^, plasticity and interanimal variability in wiring and expression. As expected, kernels from one neuron pair were more similar to each other than to kernels from other pairs (Extended Data Fig. 6f).

## Functional measurements differ from anatomy

**Fig. 3:**
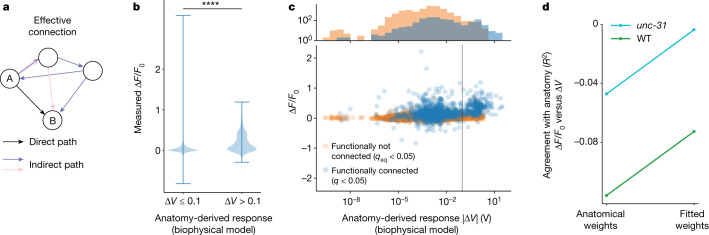
Functional measurements differ from anatomy-based predictions. **a**, Signals propagate along all paths, including indirect and recursive (coloured). Anatomical descriptions such as synapse count describe only direct paths (black). Connectome-constrained simulations are therefore used to predict signal propagation from anatomy. **b**, Pairs predicted from anatomy to have large downstream responses (Δ*V* > 0.1 V, *n* = 23, 454 pairs) tend to have stronger measured responses (larger Δ*F*/*F*_0_) than do those predicted to have small responses (Δ*V* < 0.1 V, *n* = 614 pairs). **** indicates *P* = 10^−88^, one-sided Kolmogorov–Smirnov test; whiskers indicate range. **c**, Bottom, measured downstream response (Δ*F*/*F*) versus anatomy-derived response (Δ*V*) for pairs that we observe to be functionally connected (*q* < 0.05, blue) and functionally non-connected (*q*_eq_ < 0.05, orange). Vertical grey line is (0.1 V) for comparison with **b**. Top, marginal distributions (*y* axis is log scale). Measured functionally connected pairs are enriched for predicted Δ*V* > 0.1 V, compared to functionally non-connected pairs (*P* < 0.0001, one-sided Kolmogorov–Smirnov test). **d**, Agreement of measured responses to anatomy-predicted responses is shown for WT (green) and *unc-31* (cyan) animals, either using weights and signs from anatomy, or when weights and signs are fitted optimally. Agreement is reported as *R*^2^ coefficient for the line of best fit: Δ*F*/*F*_0_ = *m*Δ*V*. Perfect agreement would be *R*^2^ = 1. Source Data

We observed an apparent contradiction with the wiring diagram—a large fraction of neuron pairs with monosynaptic (single-hop) wired connections are deemed functionally non-connected in our measurements (Fig. 2g). To further compare our measurements to anatomy, we sought to better understand what responses we should expect from the wiring diagram. Anatomical features such as synapse count are properties of only the direct (monosynaptic) connection between two neurons, but our signal propagation measurements reflect contributions from all paths through the network (Fig. 3a). To compare the two, we relied on a connectome-constrained biophysical model that predicts signal propagation from anatomy, considering all paths. We activated neurons in silico and simulated the network’s predicted response using synaptic weights from the connectome^1,6^, polarities estimated from gene expression^37^ and common assumptions about timescales and dynamics^47^.

The anatomy-derived biophysical model made some predictions that agreed with our measurements. Neuron pairs that the model predicted to have large responses (Δ*V* > 0.1) were significantly more likely to have larger measured responses than were those predicted to have little or no response (Δ*V* < 0.1) (Fig. 3b), showing agreement between structure and function. Similarly, pairs of neurons that we measured to be functionally connected (*q* < 0.05) are enriched for anatomy-predicted large responses (Δ*V* > 0.1) compared to pairs that our measurements deem functionally non-connected (*q*_eq_ < 0.05), (Fig. 3c, top).

Overall, however, there was fairly poor agreement between anatomy-based model predictions and our measurements. For example, we measured large calcium responses in neuron pairs that were predicted from anatomy to have almost no response (Fig. 3c). There was also poor agreement between anatomy-based prediction and measurement when considering the response amplitudes of all neuron pairs (Fig. 3d, *R*^2^ < 0, where an *R*^2^ of 1 would be perfect agreement).

Fundamental challenges in inferring the properties of neural connections from anatomy could contribute to the disagreement between anatomical-based model predictions and our measurements. It is challenging to infer the strength and sign of a neural connection from anatomy when many neurons send both excitatory and inhibitory signals to their postsynaptic partner^11,37^. AFD→AIY, for example, expresses machinery for inhibiting AIY through glutamate, but is excitatory owing to peptidergic signalling^48^ (Extended Data Fig. 2g). We therefore wondered whether agreement between structure and function would improve if we instead fitted the strength and sign of the wired connections to our measurements. Fitting the weights and signs, given simplifying assumptions, but forbidding new connections that do not appear in the wiring diagram, improved the agreement between the anatomical prediction and the functional measurements, although overall agreement remained poor (Fig. 3d). We therefore investigated whether additional functional connections exist beyond the connectome. We measured signal propagation in *unc-31*-mutant animals, which are defective for extrasynaptic signalling mediated by dense-core vesicles, as explained below. Although agreement was still poor, signal propagation in these animals showed better agreement with anatomy than it did in WT animals (Fig. 3d). This prompted us to consider extrasynaptic signalling further.

## Extrasynaptic signalling also drives neural dynamics

Neurons can communicate extrasynaptically by releasing transmitters, often via dense-core vesicles, that diffuse through the extracellular milieu to reach downstream neurons instead of directly traversing a synaptic cleft (Supplementary Information). Extrasynaptic signalling forms an additional layer of communication not visible from anatomy^49^ and its molecular machinery is ubiquitous in mammals^50^ and *C. elegans*^38,51,52^.

To examine the role of extrasynaptic signalling, we measured the signal propagation of *unc-31*-mutant animals defective for dense-core-vesicle-mediated release (Extended Data Fig. 7a; 18 individuals) and compared the results with those from WT animals (browsable online at https://funconn.princeton.edu). This mutation disrupts dense-core-vesicle-mediated extrasynaptic signalling of peptides and monoamines by removing UNC-31 (CAPS), a protein involved in dense-core-vesicle fusion^53^.

We expected that most signalling in the brain visible within the timescales of our measurements (30 s) would be mediated by chemical or electrical synapses and would therefore be unaffected by the *unc-31* mutation. Consistent with this, many individual functional connections that we observed in the WT case persisted in the *unc-31* mutant (Extended Data Fig. 8). But if fast dense-core-vesicle-dependent extrasynaptic signalling were present, it should be observed only in WT and not in *unc-31*-mutant individuals. Consistent with this, *unc-31* animals had a smaller proportion of functional connections than did WT animals (Extended Data Fig. 7b).

**Fig. 4:**
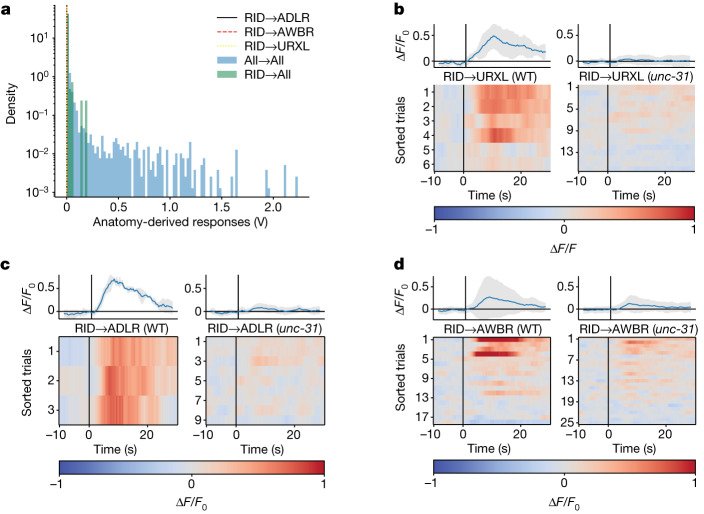
Anatomy does not capture extrasynaptic signalling from the neuron RID. **a**, ADL, AWB and URX are predicted from anatomy to have no response to RID stimulation because there is no strong anatomical path from RID to those neurons (vertical lines at or near 0 V). Their anatomy-predicted responses are shown within the distribution of anatomy-predicted responses for all neuron pairs (blue histogram), as in Fig. 3b. **b**–**d**, Activity of neurons URXL (**b**), ADLR (**c**) and AWBR (**d**) to RID stimulation, in WT and *unc-31*-mutant backgrounds. Top, mean (blue) and s.d. (shading) across trials and animals. Bottom, individual traces are sorted across trial and animal by mean response amplitude. Here, trials are shown even in cases when RID activity was not measured. Additional neurons are shown in Extended Data Fig. 7c. Source Data

We investigated the neuron RID, a cell that is thought to signal to other neurons extrasynaptically through neuropeptides, and that has only few and weak outgoing wired connections^54^. RID had dim tagRFP-T expression, so we adjusted our analysis protocol for only this neuron, as described in the Methods. Many neurons responded to RID activation (Extended Data Fig. 7c), including URX, ADL and AWB, three neuron subtypes that were predicted from anatomy to have no response (Fig. 4a). These three neurons showed strong responses in WT animals but their responses were reduced or absent in *unc-31* mutants (Fig. 4b–d), consistent with dense-core-vesicle-mediated extrasynaptic signalling. The gene expression and wiring of these neurons also suggest that peptidergic extrasynaptic signalling is producing the observed responses. All three express receptors for peptides produced by RID (NPR-4 and NPR-11 for FLP-14 and PDFR-1 for PDF-1), and no direct (monosynaptic) wiring connects RID to URX, ADL or AWB: a minimum of two hops are required from RID to URXL or AWBR, and three from RID to ADLR. These shortest paths all rely on fragile single-contact synapses that appear in only one out of the four individual connectomes^6^. We conclude that RID signals to other neurons extrasynaptically, and that this is captured by signal propagation measurements but not by anatomy.

## Extrasynaptic-dependent signal propagation screen

**Fig. 5:**
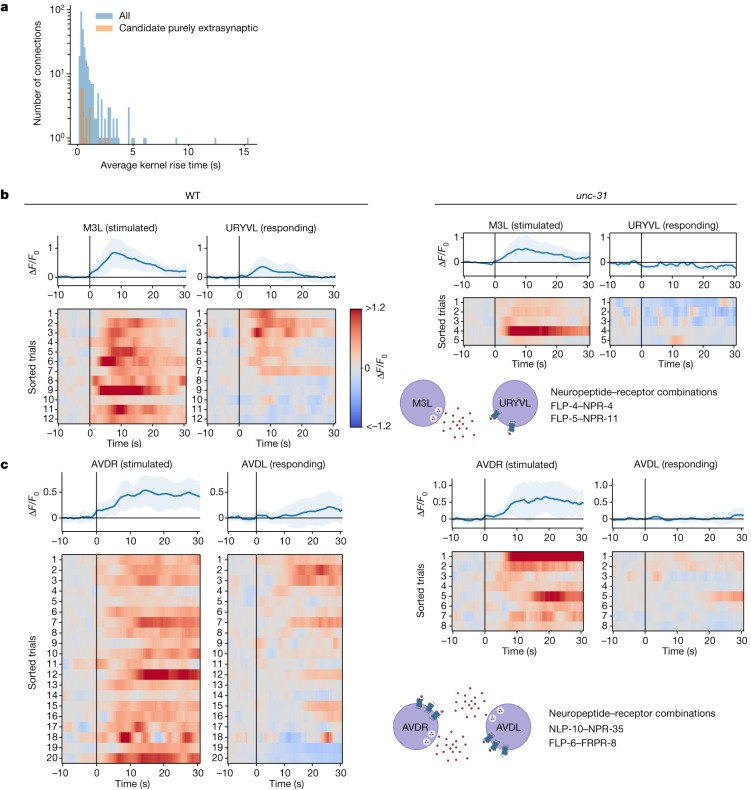
Candidate purely extrasynaptic-dependent functional connections. **a**, Distribution of signal propagation timescales. **b**,**c**, Paired responses for M3L→URYVL (**b**) and AVDR→AVDL (**c**), for WT and *unc-31* animals. *unc-31* animals do not show downstream responses to stimulation. AVDR→AVDL extrasynaptic communication is putatively mediated in autocrine loops through NLP-10→NPR-35 and FLP-6→FRPR-8 signalling. Top, average (blue) and s.d. (shading) across trials and animals. Source Data

To identify new pairs of neurons that communicate purely extrasynaptically, we performed an unbiased screen and selected for neuron pairs that had functional connections in WT animals (*q* < 0.05) but were functionally non-connected in *unc-31* mutants (*q*_eq_ < 0.05). Fifty-three pairs of neurons met our criteria (Extended Data Fig. 9), and were therefore putative candidates for purely extrasynaptic signalling. This is likely to be a lower bound because many more pairs could communicate extrasynaptically but might not appear in our screen, either because they don’t meet our statistical threshold or because they communicate through parallel paths, of which only some are extrasynaptic. Other scenarios not captured by the screen, and additional caveats, are discussed in the Supplementary Information. The timescales of signal propagation for those neuron pairs that passed our screen were similar to that of all functional connections (Fig. 5a), suggesting that in the worm, *unc-31*-dependent extrasynaptic signalling can also propagate quickly.

Neuron pair M3L→URYVL is a representative example of a purely extrasynaptic-dependent connection found from our screen. There are no direct chemical or electrical synapses between M3L and URYVL, but stimulation of M3L evokes *unc-31*-dependent calcium activity in URYVL (Fig. 5b). The majority of neuron pairs identified in our screen express peptide and receptor combinations consistent with extrasynaptic signalling^38,52^ (Supplementary Table 1). For example, M3L expresses FLP-4, which binds to the receptor NPR-4, expressed by URVYL; and FLP-5, which binds to the receptor NPR-11, also expressed by URYVL.

The bilateral neuron pair AVDR and AVDL was also identified in our screen for having purely extrasynaptic-dependent connections. AVDR and AVDL have no or only weak wired connections between them (three of four connectomes show no wired connections, and the fourth finds only a very weak gap junction), but stimulation of AVDR evoked robust *unc-31*-dependent responses in AVDL. Notably, the AVD cell type was recently predicted to have a peptidergic autocrine loop^51^ mediated by the neuropeptide–GPCR combinations NLP-10→NPR-35 and FLP-6→FRPR-8 (refs. ^38,52^) (Fig. 5c). The bilateral extrasynaptic signalling that we observe is consistent with this prediction because two neurons that express the same autocrine signalling machinery can necessarily signal to one another. AVD was also predicted to be among the top 25 highest-degree ‘hub’ nodes in a peptidergic network based on gene expression^51^, and, in agreement, AVD is highly represented among hits in our screen (Extended Data Fig. 9b).

## Signal propagation predicts spontaneous activity

**Fig. 6:**
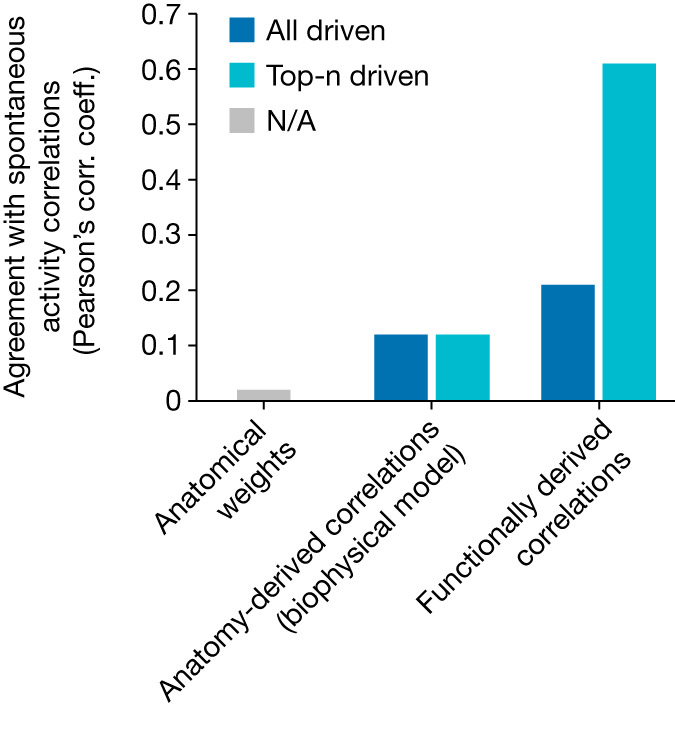
Measured signal propagation better predicts spontaneous activity than anatomy does. Agreement (as Pearson’s correlation (corr.) coefficient (coeff.)) between the correlation matrix of spontaneous activity recorded from an immobilized animal and various predictions of those correlations, including: the bare anatomical weight matrix (synapse counts) (left); correlations predicted by the anatomy-derived biophysical model (middle); and correlations functionally derived from the measured signal propagation kernels (right). Anatomy-derived and functionally derived correlations are calculated by driving activity in silico in all neurons (dark blue) or only an optimal subset of top-n neurons (light blue). NA, not applicable. Source Data

A key motivation for mapping neural connections is to understand how they give rise to collective neural dynamics. We tested the ability of our signal propagation map to predict worms’ spontaneous activity, and compared this to predictions from anatomy (Fig. 6). Spontaneous activity was measured in immobilized worms lacking optogenetic actuators under bright imaging conditions. A matrix of bare anatomical weights (synapse counts) was a poor predictor of the correlations of spontaneous activity (left bar, Fig. 6), consistent with previous reports^27,41^. The connectome-constrained biophysical model from Fig. 3 better predicted spontaneous activity correlations (middle bars, Fig. 6; described in the Methods)—as we would expect because it considers all anatomical paths through the network—but it still performed fairly poorly. Predictions based on our functional measurements of signal propagation kernels (right bars, Fig. 6) performed best of all at predicting spontaneous activity correlations. To generate predictions of correlations either from the biophysical model or from our functional kernel measurements required the activity of a set of neurons to be driven in silico. For the biophysical model, driving all neurons was optimal, but for the kernel-based predictions, driving a specific set of six neurons (‘top-n’) markedly improved performance. We conclude that functionally derived predictions based on our measured signal propagation kernels better agree with spontaneous activity than do either a bare description of anatomical weights or an established model constrained by the connectome, and that some subsets of neurons make outsized contributions to driving spontaneous dynamics. The kernel-based simulation (interactive version at https://funsim.princeton.edu) outperforms other models of neural dynamics presumably for two reasons: first, it extracts all relevant parameters directly from the measured kernels, thereby avoiding the need for many assumptions; and second, it captures extrasynaptic signalling not visible from anatomy.

## Discussion

Signal propagation in *C. elegans* measured by neural activation differs from model predictions based on anatomy, in part because anatomy does not account for wireless connections such as the extrasynaptic release of neuropeptides^49^.

By directly evoking calcium activity on a timescale of seconds, extrasynaptic signalling serves a functional role similar to that of classical neurotransmitters and contributes to neural dynamics. This role is in addition to its better-characterized role in modulating neural excitability over longer timescales.

Peptidergic extrasynaptic signalling relies on diffusion and therefore may be uniquely well suited to *C. elegans*’ small size. Mammals also express neuropeptides and receptors, including in the cortex^50^, but their larger brains might limit the speed, strength or spatial extent of peptidergic extrasynaptic signalling.

Plasticity, neuromodulation, neural-network state, experience dependence and other longer-timescale effects might contribute to variability in our measured responses or to discrepancies between anatomical and functional descriptions of the *C. elegans* network. A future direction will be to search for latent connections that might become functional only during certain internal states.

Our signal propagation map provides a lower bound on the number of functional connections (Supplementary Information). Our measurements required a trade-off between the animal’s health and its transgenic load. To express the necessary transgenes, we generated a strain that is not behaviourally wild type; its signal propagation might therefore also differ from the wild type. To probe nonlinearities and multi-neuron interactions in the network, future measurements are needed of the network’s response to simultaneous stimulation of multiple neurons.

Our signal propagation map reports effective connections, not direct connections. Effective connections are useful for the circuit-level questions that motivate our work, such as how a stimulus in one part of the network drives activity in another. Direct connections are suited for questions of gene expression, development and anatomy, but less so for network function. For example, a direct connection between two neurons could be slow or weak, but might overlook a fast and strong effective connection via other paths through the network.

We used a connectome-constrained biophysical model to provide additional evidence to support our claim that measured signal propagation differs from expectations based on anatomy. The model relies on assumptions of timescales, nonlinearities and other parameters that, if incorrect, would contribute to the observed disagreement between anatomy and function. But even without any biophysical model, discrepancies between anatomy and function are apparent; for example, in pairs of neurons with synaptic connections that are functionally non-connected (Fig. 2g), and in strong functional connections between RID and other neurons that have only weak, variable and indirect synaptic connections (Fig. 4). The challenge of confidently constraining model parameters from anatomy highlights the need for functional measurements, like the ones performed here. These functional measurements fill in fundamental gaps in the translation from anatomical connectome to neural activity. An alternative approach for comparing structure and function would be to infer properties of direct connections from the measured effective connections^55^, but this might require a higher signal-to-noise ratio than our current measurements.

The signal propagation atlas presented here informs structure–function investigations at both the circuit and the network level, and enables more accurate brain-wide simulations of neural dynamics. The finding that extrasynaptic peptidergic signalling, which is invisible to anatomy, evokes neural dynamics in *C. elegans* will inform ongoing discussions about how to characterize other brains in more detail and on a larger scale.

## Methods

### Worm maintenance

**Extended Data Fig. 1:**
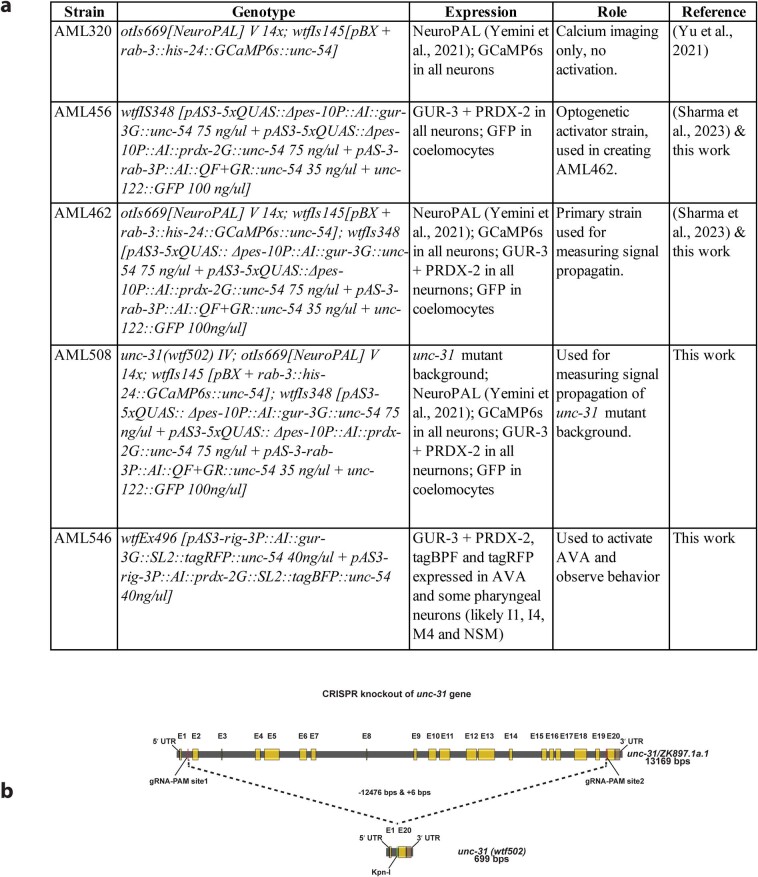
Strains. **a**, Table of strains used in this work. **b**, Schematic of CRISPR knockout of *unc-31*.

*C. elegans* were stored in the dark, and only minimal light was used when transferring worms or mounting worms for experiments. Strains generated in this study (Extended Data Fig. 1a) have been deposited in the Caenorhabditis Genetics Center (CGC), University of Minnesota, for public distribution. Hermaphrodites were used in this study.

### Transgenics

We generated a transgenic worm for interrogating signal propagation, TWISP (AML462), which has been described in more detail previously^23^. This strain expresses the calcium indicator GCaMP6s in the nucleus of each neuron; a purple-light-sensitive optogenetic protein system (GUR-3 and PRDX-2) in each neuron; and multiple fluorophores of various colours from the NeuroPAL^27^ system, also in the nucleus of neurons. We also used a QF-hGR drug-inducible gene-expression strategy to turn on the gene expression of optogenetic actuators only later in development. To create this strain, we first generated an intermediate strain, AML456, by injecting a plasmid mix (75 ng μl^−1^ pAS3-5xQUAS::Δ pes-10P::AI::gur-3G::unc-54 + 75 ng μl^−1^ pAS3-5xQUAS::Δ pes-10P::AI::prdx-2G::unc-54 + 35 ng μl^−1^ pAS-3-rab-3P::AI::QF+GR::unc-54 + 100 ng μl^−1^ unc-122::GFP) into CZ20310 worms, followed by UV integration and six outcrosses^56,57^. The intermediate strain, AML456, was then crossed into the pan-neuronal GCaMP6s calcium-imaging strain, with NeuroPAL, AML320 (refs. ^23,27,58^).

Animals exhibited decreased average locomotion compared to the WT (mean speeds of 0.03 mm s^−1^ off drug and 0.02 mm s^−1^ on drug compared to the mean of 0.15 mm s^−1^ in WT animals^23^), as expected for NeuroPAL GCaMP6s strains, which are also reported to be overall less active (around 0.09 mm s^−1^ during only forward locomotion)^27^.

An *unc-31*-mutant background with defects in the dense-core-vesicle-release pathway was used to diminish wireless signalling^53^. We created an *unc-31*-knockout version of our functional connectivity strain by performing CRISPR–Cas9-mediated genome editing on AML462 using a single-strand oligodeoxynucleotide (ssODN)-based homology-dependent repair strategy^59^. This approach resulted in strain AML508 (unc-31 (wtf502) IV; otIs669 (NeuroPAL) V 14x; wtfIs145 (30 ng μl^−1^ *pBX* + 30 ng μl^−1^ *rab-3::his-24::GCaMP6s::unc-54*); *wtfIs348* (75 ng μl^−1^ *pAS3-5xQUAS::Δ pes-10P::AI::gur-3G::unc-54* + 75 ng μl^−1^ *pAS3-5xQUAS::Δ pes-10P::AI::prdx-2G::unc-54* + 35 ng μl^−1^ *pAS-3-rab-3P::QF+GR::unc-54* + 100 ng μl^−1^ *unc-122::GFP*)).

CRISPR–Cas-9 editing was carried out as follows. Protospacer adjacent motif (PAM) sites (denoted in upper case) were selected in the first intron (gagcuucgcaauguugacucCGG) and the last intron (augguacauuggguccguggCGG) of the *unc-31* gene (*ZK897.1a.1*) to delete 12,476 out of 13,169 bp (including the 5′ and 3′ untranslated regions) and 18 out of 20 exons from the genomic locus, while adding 6 bp (GGTACC) for the Kpn-I restriction site (Extended Data Fig. 1b). Alt-R S.p. Cas9 Nuclease V3, Alt-R-single guide RNA (sgRNA) and Alt-R homology-directed repair (HDR)-ODN were used (IDT). We introduced the Kpn-I restriction site, denoted in upper case (gacccagcgaagcaaggatattgaaaacataagtacccttgttgttgtgtGGTACCccacggacccaatgtaccatattttacgagaaatttataatgttcagg) into our repair oligonucleotide to screen and confirm the deletion by PCR followed by restriction digestion. sgRNA and HDR ssODNs were also synthesized for the *dpy-10* gene as a reporter, as described previously^59^. An injection mix was prepared by sequentially adding Alt-R S.p. Cas9 Nuclease V3 (1 μl of 10 μg μl^−1^), 0.25 μl of 1 M KCL, 0.375 μl of 200 mM HEPES (pH 7.4), sgRNAs for *unc-31* (1 μl each for both sites) and 0.75 μl for *dpy-10* from a stock of 100 μM, ssODNs (1 μl for *unc-31* and 0.5 μl for *dpy-10* from a stock of 25 μM) and nuclease-free water to a final volume of 10 μl in a PCR tube, kept on ice. The injection mix was then incubated at 37 °C for 15 min before it was injected into the germline of AML462 worms. Progenies from plates showing roller or dumpy phenotypes in the F_1_ generation after injection were individually propagated and screened by PCR and Kpn-I digestion to confirm deletion. Single-worm PCR was carried out using GXL-PRIME STAR taq-Polymerase (Takara Bio) and the Kpn-1-HF restriction enzyme (NEB). Worms without a roller or dumpy phenotype and homozygous for deletion were confirmed by Sanger sequencing fragment analysis.

To cross-validate GUR-3/PRDX-2-evoked behaviour responses, we generated the transgenic strain AML546 by injecting a plasmid mix (40 ng μl^−1^ pAS3-rig-3P::AI::gur-3G::SL2::tagRFP::unc-54 + 40 ng μl^−1^ pAS3-rig-3P::AI::prdx-2G::SL2::tagBFP::unc-54) into N2 worms to generate a transient transgenic line expressing GUR-3/PRDX-2 in AVA neurons.

### Cross-validation of GUR-3/PRDX-2-evoked behaviour

**Extended Data Fig. 2:**
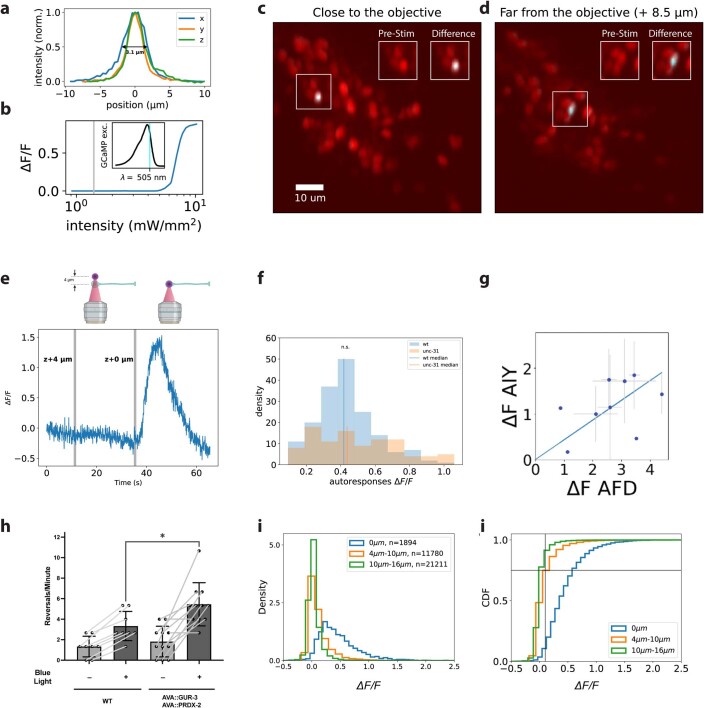
Characterization of two-photon optogenetic stimulation and evoked response. **a**, Two-photon (2p) stimulation spot size (point-spread function). **b**, Imaging excitation wavelength and intensity were chosen to avoid GUR-3/PRDX-2 activation. GCaMP response to 500 nm activation of GUR-3/PRDX-2 expressing neuron as reported in^24^. Vertical grey line indicates light intensity typically used for calcium imaging in present work. Inset: GCaMP6 excitation spectra from^26^. Vertical cyan line indicates 505-nm imaging excitation wavelength used in present work. **c**,**d**, A neuron near (**c**) and a neuron far (**d**) from the objective are photobleached to demonstrate targeted illumination. tagRFP-T is photobleached by 2p stim (20 s illumination, 200 μW, 500 kHz repetition rate, 3.1 μm diameter FWHM spot). Difference image shows tagRFP-T fluorescence merged with a false-colour blue-green image to reveal change in intensity after targeted illumination. Only targeted neuron and not nearby neurons appear photobleached. Insets shows zoomed-in image of the targeted neuron’s original tagRFP-T intensity (left) and difference image (right). Laser power was chosen to avoid saturated bleaching. For a sense of scale, C. elegans interneuron cell somas are roughly 4 microns in diameter. **e**, In vivo demonstration of 2p effective spot size. Activity from a neuron expressing GUR-3/PRDX-2 and GCaMP6s is shown in response to a 300-ms 2p stimulation delivered at *t* = 11 s,4 μm beyond the ≈ 3.5 μm diameter soma on the optical axis (z), and at *t* = 35 s, centred on the soma (*t* = 35 s). Only on-target soma stimulation evokes a transient, called an “autoresponse”. A stimulus artefact at *t* = 35 s is visible because no smoothing or filtering is applied to this trace. Schematic via BioRender. **f**, Distribution of autoresponses under typical stimulus conditions (1.2 mW, 500 kHz; 0.5 s for WT, 0.3 s for *unc-31*). Autoresponses are required for inclusion. **g**, Measured calcium response of neuron AIY to optogenetic stimulation of AFD. Compare to figure 4b in ref. ^48^. A variety of stimulus durations was used to generate autoresponses of different amplitudes (n = 1 (0.1 s), n = 2 (0.15 s), n = 1 (0.2 s), n = 3 (0.25 s), n = 3 (0.3 s), n = 3 (0.35 s), n = 6 (0.4 s), n = 2 (0.45 s), n = 4 (0.5 s) cross-hairs indicate s.d. **h**, Blue light evoked more reversals in animals expressing GUR-3/PRDX-2 in AVA (n = 11 animals) than WT (n = 8 animals). ~480 nm peaked light was delivered to freely moving animals. Unpaired *t*-test, *p* = 0.025. Bars show mean and s.d. **i**,**j**, Probability density (**i**) and CDF (**j**) of evoked calcium responses in a 30-s post-stimulus window for the targeted neuron (0 μm) or for neurons different distances away. Autoresponses are required. Cross-hairs in **j**, 75% cumulative distribution at Δ*F*/*F*_0_ = 0.1. Source Data

Optogenetic activation of AVA neurons using traditional channelrhodopsins (for example, Chrimson) leads to reversals^45,60^. We used worms expressing GUR-3/PRDX-2 in AVA neurons (AML564) to show that GUR-3/PRDX-2 elicits a similar behavioural response. We illuminated freely moving worms with blue light from an LED (peaked at 480 nm, 2.3 mW mm^−^^2^) for 45 s. We compared the number of onsets of reversals in that period of time with a control in which only dim white light was present, as well as with the results of the same assay performed on N2 worms. Animals with GUR-3/PRDX-2 in AVA (*n* = 11 animals) exhibited more blue-light-evoked reversals per minute than did WT animals (*n* = 8 animals) (Extended Data Fig. 2h).

### Dexamethasone treatment

To increase the expression of optogenetic proteins while avoiding arrested development, longer generation time and lethality, a drug-inducible gene-expression strategy was used. Dexamethasone (dex) activates QF-hGR to temporally control the expression of downstream targets^61^, in this case the optogenetic proteins in the functional connectivity imaging strains AML462 and AML508. Dex-NGM plates were prepared by adding 200 μM of dex in dimethyl sulfoxide (DMSO) just before pouring the plate. For dex treatment, L2/L3 worms were transferred to overnight-seeded dex-NGM plates and further grown until worms were ready for imaging. More details of the dex treatment are provided below.

We prepared stock solution of 100 mM dex by dissolving 1 g dexamethasone (D1756, Sigma-Aldrich) in 25.5 ml DMSO (D8418, Sigma-Aldrich). Stocks were then filter-sterilized, aliquoted, wrapped in foil to prevent light and stored at −80 °C until needed. The 200-μM dex-NGM plates were made by adding 2 ml of 100 mM dex stock in 1 l NGM-agar medium, while stirring, 5 min before pouring the plate. Dex plates were stored at 4 °C for up to a month until needed.

### Preparation of worms for imaging

Worms were individually mounted on 10% agarose pads prepared with M9 buffer and immobilized using 2 μl of 100-nm polystyrene beads solution and 2 μl of levamisole (500 μM stock). This concentration of levamisole, after dilution in the polystyrene bead solution and the agarose pad water, largely immobilized the worm while still allowing it to slightly move, especially before placing the coverslip. Pharyngeal pumping was observed during imaging.

### Overview of the imaging strategy

We combined whole-brain calcium imaging through spinning disk single-photon confocal microscopy^62,63^ with two-photon^64^ targeted optogenetic stimulation^65^, each with their own remote focusing system, to measure and manipulate neural activity in an immobilized animal (Fig. 1a). We performed calcium imaging, with excitation light at a wavelength and intensity that does not elicit photoactivation of GUR-3/PRDX-2 (ref. ^66^) (Extended Data Fig. 2b). We also used genetically encoded fluorophores from NeuroPAL expressed in each neuron^27^ to identify neurons consistently across animals (Fig. 1c).

### Multi-channel imaging and neural identification

Volumetric, multi-channel imaging was performed to capture images of the following fluorophores in the NeuroPAL transgene: mtagBFP2, CyOFP1.5, tagRFP-T and mNeptune2.5 (ref. ^27^). Light downstream of the same spinning disk unit used for calcium imaging travelled on an alternative light path through channel-specific filters mounted on a mechanical filter wheel, while mechanical shutters alternated illumination with the respective lasers, similar to a previously described method^58^. Channels were as follows: mtagBFP2 was imaged using a 405-nm laser and a Semrock FF01-440/40 emission filter; CyOFP1.5 was imaged using a 505-nm laser and a Semrock 609/54 emission filter; tagRFP-T was imaged using a 561-nm laser and a Semrock 609/54-nm emission filter; and mNeptune2.5 was imaged using a 561-nm laser and a Semrock 732/68-nm emission filter.

After the functional connectivity recording was complete, neuron identities were manually assigned by comparing each neuron’s colour, position and size to a known atlas. Some neurons are particularly hard to identify in NeuroPAL and are therefore absent or less frequently identified in our recordings. Some neurons have dim tagRFP-T expression, which makes it difficult for the neuron segmentation algorithm to find them and, therefore, to extract their calcium activity. These neurons include, for example, AVB, ADF and RID. RID’s distinctive position and its expression of CyOFP allowed us nevertheless to manually target it optogenetically. Neurons in the ventral ganglion are hard to identify because it appears as very crowded when viewed in the most common orientation that worms assume when mounted on a microscope slide. Neurons in the ventral ganglion are therefore sometimes difficult to distinguish from one another, especially for dimmer neurons such as the SIA, SIB and RMF neurons. In our strain, the neurons AWCon and AWCoff were difficult to tell apart on the basis of colour information.

### Volumetric image acquisition

Neural activity was recorded at whole-brain scale and cellular resolution through continuous acquisition of volumetric images in the red and green channels with a spinning disk confocal unit and using LabView software (https://github.com/leiferlab/pump-probe-acquisition/tree/pp), similarly to a previous study^67^, with a few upgrades. The imaging focal plane was scanned through the brain of the worm remotely using an electrically tunable lens (Optotune EL-16-40-TC) instead of moving the objective. The use of remote focusing allowed us to decouple the *z*-position of the imaging focal plane and that of the optogenetics two-photon spot (described below).

Images were acquired by an sCMOS camera, and each acquired image frame was associated to the focal length of the tunable lens (*z*-position in the sample) at which it was acquired. To ensure the correct association between frames and *z*-position, we recorded the analogue signal describing the focal length of the tunable lens at time points synchronous with a trigger pulse output by the camera. By counting the camera triggers from the start of the recording, the *z*-positions could be associated to the correct frame, bypassing unknown operating-system-mediated latencies between the image stream from the camera and the acquisition of analogue signals.

In addition, real-time ‘pseudo’-segmentation of the neurons (described below) required the ability to separate frames into corresponding volumetric images in real time. Because the *z*-position was acquired at a low sample rate, splitting of volumes on the basis of finite differences between successive *z*-positions could lead to errors in assignment at the edge of the *z*-scan. An analogue OP-AMP-based differentiator was used to independently detect the direction of the *z*-scan in hardware.

### Calcium imaging

Calcium imaging was performed in a single-photon regime with a 505-nm excitation laser through spinning disk confocal microscopy, at 2 vol s^−1^. For functional connectivity experiments, an intensity of 1.4 mW mm^−2^ at the sample plane was used to image GCaMP6s, well below the threshold needed to excite the GUR-3/PRDX-2 optogenetic system^24^. We note that at this wavelength and intensity, animals exhibited very little spontaneous calcium activity.

For certain analyses (Fig. 6), recordings with ample spontaneous activity were desired. In those cases, we increased the 505-nm intensity sevenfold, to approximately 10 mW mm^−2^, and recorded from AML320 strains that lacked exogenous GUR-3/PRDX-2 to avoid potential widespread neural activation. Under these imaging conditions, we observed population-wide slow stereotyped spontaneous oscillatory calcium dynamics, as previously reported^35,68^.

### Extraction of calcium activity from the images

Calcium activity was extracted from the raw images by using Python libraries implementing optimized versions of a previously described algorithm^69^, available at https://www.github.com/leiferlab/pumpprobe, https://www.github.com/leiferlab/wormdatamodel, https://www.github.com/leiferlab/wormneuronsegmentation-c and https://www.github.com/leiferlab/wormbrain.

The positions of neurons in each acquired volume were determined by computer vision software implemented in C++. This software was greatly optimized to identify neurons in real time, to also enable closed-loop targeting and stimulus delivery (as described in ‘Stimulus delivery and pulsed laser’). Two design choices made this algorithm considerably faster than previous approaches. First, a local maxima search was used instead of a slower watershed-type segmentation. The nuclei of *C. elegans* neurons are approximately spheres and so they can be identified and separated by a simple local maxima search. Second, we factorized the three-dimensional (3D) local maxima search into multiple two-dimensional (2D) local maxima searches. In fact, any local maximum in a 3D image is also a local maximum in the 2D image in which it is located. Local maxima were therefore first found in each 2D image separately, and then candidate local maxima were discarded or retained by comparing them to their immediate surroundings in the other planes. This makes the algorithm less computationally intensive and fast enough to also be used in real time. We refer to this type of algorithm as ‘pseudo’-segmentation because it finds the centre of neurons without fully describing the extent and boundaries of each neuron.

After neural locations were found in each of the volumetric images, a nonrigid point-set registration algorithm was used to track their locations across time, matching neurons identified in a given 3D image to the neurons identified in a 3D image chosen as reference. Even worms that are mechanically immobilized still move slightly and contract their pharynx, thereby deforming their brain and requiring the tracking of neurons. We implemented in C++ a fast and optimized version of the Dirichelet–Student’s-*t* mixture model (DSMM)^70^.

### Calcium pre-processing

The GCaMP6s intensity extracted from the images undergoes the following pre-processing steps. (1) Missing values are interpolated on the basis of neighbouring time points. Missing values can occur when a neuron cannot be identified in a given volumetric image. (2) Photobleaching is removed by fitting a double exponential to the baseline signal. (3) Outliers more than 5 standard deviations away from the average are removed from each trace. (4) Traces are smoothed using a causal polynomial filtering with a window size of 6.5 s and polynomial order of 1 (Savitzky–Golay filters with windows completely ‘in the past’; for example, obtained with scipy.signal.savgol_coeffs(window_length=13, polyorder=1, pos=12)). This type of filter with the chosen parameters is able to remove noise without smearing the traces in time. Note that when fits are performed (for example, to calculate kernels), they are always performed on the original, non-smoothed traces. (5) Where Δ*F*/*F*_0_ of responses is used, *F*_0_ is defined as the value of *F* in a 30-s interval before the stimulation time and Δ*F* ≡ *F* − *F*_0_. In Fig. 2a, for example, ${ < \Delta F/{F}_{0} > }_{t}$ refers to the mean over a 30-s post-stimulus window.

### Stimulus delivery and pulsed laser

For two-photon optogenetic targeting, we used an optical parametric amplifier (OPA; Light Conversion ORPHEUS) pumped by a femtosecond amplified laser (Light Conversion PHAROS). The output of the OPA was tuned to a wavelength of 850 nm, at a 500 kHz repetition rate. We used temporal focusing to spatially restrict the size of the two-photon excitation spot along the microscope axis. A motorized iris was used to set its lateral size. For temporal focusing, the first-order diffraction from a reflective grating, oriented orthogonally to the microscope axis, was collected (as described previously^71^) and travelled through the motorized iris, placed on a plane conjugate to the grating. To arbitrarily position the two-photon excitation spot in the sample volume, the beam then travelled through an electrically tunable lens (Optotune EL-16-40-TC, on a plane conjugate to the objective), to set its position along the microscope axis, and finally was reflected by two galvo-mirrors to set its lateral position. The pulsed beam was then combined with the imaging light path by a dichroic mirror immediately before entering the back of the objective.

Most of the stimuli were delivered automatically by computer control. Real-time computer vision software found the position of the neurons for each volumetric image acquired, using only the tagRFP-T channel. To find neural positions, we used the same pseudo-segmentation algorithm described above. The algorithm found neurons in each 2D frame in around 500 μs as the frames arrived from the camera. In this way, locations for all neurons in a volume were found within a few milliseconds of acquiring the last frame of that volume.

Every 30 s, a random neuron was selected among the neurons found in the current volumetric image, on the basis of only its tagRFP-T signal. After galvo-mirrors and the tunable lens set the position of the two-photon spot on that neuron, a 500-ms (300-ms for the *unc-31*-mutant strain) train of light pulses was used to optogenetically stimulate that neuron. The duration of stimulus illumination for the *unc-31*-mutant strain was selected to elicit calcium transients in stimulated neurons with a distribution of amplitudes such that the maximum amplitude was similar to those in WT-background animals, (Extended Data Fig. 2f). The output of the laser was controlled through the external interface to its built-in pulse picker, and the power of the laser at the sample was 1.2 mW at 500 kHz. Neuron identities were assigned to stimulated neurons after the completion of experiments using NeuroPAL^27^.

To probe the AFD→AIY neural connection, a small set of stimuli used variable pulse durations from 100 ms to 500 ms in steps of 50 ms selected randomly to vary the amount of optogenetic activation of AFD.

In some cases, neurons of interest were too dim to be detected by the real-time software. For those neurons of interest, additional recordings were performed in which the neuron to be stimulated was manually selected on the basis of its colour, size and position. This was the case for certain stimulations of neurons RID and AFD.

### Characterization of the size of the two-photon excitation spot

The lateral (*xy*) size of the two-photon excitation spot was measured with a fluorescent microscope slide, and the axial (*z*) size was measured using 0.2-nm fluorescent beads (Suncoast Yellow, Bangs Laboratories), by scanning the *z*-position of the optogenetic spot while maintaining the imaging focal plane fixed (Extended Data Fig. 2a).

We further tested our targeted stimulation in two ways: selective photobleaching and neuronal activation. First, we targeted individual neurons at various depths in the worm’s brain, and we illuminated them with the pulsed laser to induce selective photobleaching of tagRFP-T. Extended Data Fig. 2c,d shows how our two-photon excitation spot selectively targets individual neurons, because it photobleaches tagRFP-T only in the neuron that we decide to target, and not in nearby neurons. To faithfully characterize the spot size, we set the laser power such that the two-photon interaction probability profile of the excitation spot would not saturate the two-photon absorption probability of tagRFP-T. Second, we showed that our excitation spot is restricted along the *z*-axis by targeting a neuron and observing its calcium activity. When the excitation was directed at the neuron but shifted by 4 μm along *z*, the neuron showed no activation. By contrast, the neuron showed activation when the spot was correctly positioned on the neuron (Extended Data Fig. 2e). To further show that our stimulation is spatially restricted to an individual neuron more broadly throughout our measurements, we show that stimulations do not elicit responses in most of the close neighbours of the targeted neurons (Extended Data Fig. 2i and Supplementary Information).

### Inclusion criteria

Stimulation events were included for further analysis if they evoked a detectable calcium response in the stimulated neuron (autoresponse). A classifier determined whether the response was detected by inspecting whether the amplitude of both the Δ*F*/*F*_0_ transient and its second derivative exceeded a pair of thresholds. The same threshold values were applied to every animal, strain, neuron and stimulation event, and were originally set to match the human perception of a response above noise. Stimulation events that did not meet both thresholds for a contiguous 4 s were excluded. The RID responses shown in Fig. 4 and Extended Data Fig. 7c are an exception to this policy. RID is visible on the basis of its CyOFP expression, but its tagRFP-T expression is too dim to consistently extract calcium signals. Therefore, in Fig. 4 and Extended Data Fig. 7c (but not in other figures, such as Fig. 2), downstream neurons’ responses to RID stimulation were included even in cases in which it was not possible to extract a calcium-activity trace in RID.

Neuron traces were excluded from analysis if a human was unable to assign an identity or if the imaging time points were absent in a contiguous segment longer than 5% of the response window owing to imaging artefacts or tracking errors. A different policy applies to dim neurons of interest that are not automatically detected by the pseudo-segmentation algorithm in the 3D image used as reference for the point-set registration algorithm. In those cases, we manually added the position of those neurons to the reference 3D image. If these ‘added’ neurons are automatically detected in most of the other 3D images, then a calcium activity trace can be successfully produced by the DSMM nonrigid registration algorithm, and is treated as any other trace. However, if the ‘added’ neurons are too dim to be detected also in the other 3D images and the calcium activity trace cannot be formed for more than 50% of the total time points, the activity trace for those neurons is extracted from the neuron’s position as determined from the position of neighbouring neurons. In the analysis code, we refer to these as ‘matchless’ traces, because the reference neuron is not matched to any detected neuron in the specific 3D image, but its position is just transformed according to the DSMM nonrigid deformation field. In this way, we are able to recover the calcium activity of some neurons whose tagRFP-T expression is otherwise too dim to be reliably detected by the pseudo-segmentation algorithm. Responses to RID stimulation shown in Fig. 4 and Extended Data Fig. 7c are an exception to this policy. In these cases, the activity of any neuron for which there is not a trace for more than 50% of the time points is substituted with the corresponding ‘matchless’ trace, and not just for the manually added neurons. This is important to be able to show responses of neurons such as ADL, which have dim tagRFP-T expression. In the RID-specific case, to exclude responses that become very large solely because of numerical issues in the division by the baseline activity owing to the dim tagRFP-T, we also introduce a threshold excluding Δ*F*/*F* > 2.

Kernels were computed only for stimulation-response events for which the automatic classifier detected responses in both the stimulated and the downstream neurons. If the downstream neuron did not show a response, we considered the downstream response to be below the noise level and the kernel to be zero.

### Statistical analysis

We used two statistical tests to identify neuron pairs that under our stimulation and imaging conditions can be deemed ‘functionally connected’, ‘functionally non-connected’ or for which we lack the confidence to make either determination. Both tests compare observed calcium transients in each downstream neuron to a null distribution of transients recorded in experiments lacking stimulation.

To determine whether a pair of neurons can be deemed functionally connected, we calculated the probability of observing the measured calcium response in the downstream neuron given no neural stimulation. We used a two-sided Kolmogorov–Smirnov test to compare the distributions of the downstream neuron’s Δ*F*/*F*_0_ amplitude and its temporal second derivative from all observations of that neuron pair under stimulation to the empirical null distributions taken from control recordings lacking stimulation. *P* values were calculated separately for Δ*F*/*F*_0_ and its temporal second derivative, and then combined using Fischer’s method to report a single fused *P* value for each neuron pair. Finally, to account for the large number of hypotheses tested, a false discovery rate was estimated. From the list of *P* values, each neuron was assigned a *q* value using the Storey–Tibshirani method^40^. *q* values are interpreted as follows: when considering an ensemble of putative functional connections of *q* values all less than or equal to *q*_*c*_, an approximately *q*_*c*_ fraction of those connections would have appeared in a recording that lacked any stimulation.

To explicitly test whether a pair of neurons are functionally not connected, taking into account the amplitude of the response, their reliability, the number of observations and multiple hypotheses, we also computed equivalence *P*_eq_ and *q*_eq_ values. This assesses the confidence of a pair not being connected. We test whether our response is equivalent to what we would expect from our control distribution using the two one-sided t-test (TOST)^72^. We computed *P*_eq_ values for Δ*F*/*F*_0_ and its temporal second derivative for a given pair being equivalent to the control distributions within an ${\epsilon }=1.2{\sigma }_{\Delta F/{F}_{0},{\partial }_{t}^{2}}$. Here, ${\sigma }_{\Delta F/{F}_{0},{\partial }_{t}^{2}}$ is the standard deviation of the corresponding control distribution. We then combined the two *P*_eq_ values into a single one with the Fisher method and computed *q*_eq_ values using the Storey–Tibshirani method^40^. Note that, different from the regular *P* values described above, the equivalence test relies on the arbitrary choice of *ϵ*, which defines when we call two distributions equivalent. We chose a conservative value of *ϵ* = 1.2*σ*.

We note that the statistical framework is stringent and a large fraction of measured neuron pairs fail to pass either statistical test.

### Measuring path length through the synaptic network

To find the minimum path length between neurons in the anatomical network topology, we proceeded iteratively. We started from the original binary connectome and computed the map of strictly two-hop connections by looking for pairs of neurons that are not connected in the starting connectome (the actual anatomical connectome at the first step) but that are connected through a single intermediate neuron. To generate the strictly three-hop connectome, we repeated this procedure using the binary connectome including direct and two-hop connections, as the starting connectome. This process continued iteratively to generate the strictly *n*-hop connectome.

In the anatomical connectome (the starting connectome for the first step in the procedure above), a neuron was considered to be directly anatomically connected if the connectomes of any of the four L4 or adult individuals in refs. ^1^ and ^6^ contained at least one synaptic contact between them. Note that this is a permissive description of anatomical connections, as it considers even neurons with only a single synaptic contact in only one individual to be connected.

### Fitting kernels

Kernels *k*_*ij*_(*t*) were defined as the functions to be convolved with the activity Δ*F*_*j*_ of the stimulated neuron to obtain the activity Δ*F*_*i*_ of a responding neuron *i*, such that $\Delta {F}_{i}(t)=({k}_{ij}\ast \Delta {F}_{j})(t)$. To fit kernels, each kernel *k*(*t*) was parametrized as a sum of convolutions of decaying exponentials

1 $$k(t)=\sum _{m}{c}_{m}(\theta (t){e}^{-{\gamma }_{m,0}t})\ast (\theta (t){e}^{-{\gamma }_{m,1}t})\ast ...,$$

where the indices *i*, *j* are omitted for clarity and *θ* is the Heaviside function. This parametrization is exact for linear systems, and works as a description of causal signal transmission also in nonlinear systems. Note that increasing the number of terms in the successive convolutions does not lead to overfitting, as would occur by increasing the degree of a polynomial. Overfitting could occur by increasing the number of terms in the sum, which in our fitting is constrained to be a maximum of 2. The presence of two terms in the sum allows the kernels to represent signal transmission with saturation (with *c*_0_ and *c*_1_ of opposite signs) and assume a fractional-derivative-like shape.

The convolutions are performed symbolically. The construction of kernels as in equation (1) starts from a symbolically stored, normalized decaying exponential kernel with a factor $A,A{\gamma }_{0}\theta (t){e}^{-{\gamma }_{0}t}$. Convolutions with normalized exponentials ${\gamma }_{n}\theta (t){e}^{-{\gamma }_{n}t}$ are performed sequentially and symbolically, taking advantage of the fact that successive convolutions of exponentials always produce a sum of functions in the form ∝ *θ*(*t*)*t*^*n*^*e*^−*γt*^. Once rules are found to convolve an additional exponential with a function in that form, any number of successive convolution can be performed. These rules are as follows:

1. If the initial term is a simple exponential with a given factor (not necessarily just the normalization *γ*) ${c}_{i}\theta (t){e}^{-{\gamma }_{i}t}$ and *γ*_*i*_ ≠ *γ*_*n*_, then the convolution is
  2 $${c}_{i}\theta (t){e}^{-{\gamma }_{i}t}\ast {\gamma }_{n}\theta (t){e}^{-{\gamma }_{n}t}={c}_{\mu }\theta (t){e}^{-{\gamma }_{\mu }t}+{c}_{\nu }\theta (t){e}^{-{\gamma }_{\nu }t},$$
  with $${c}_{\mu }=\frac{{c}_{i}{\gamma }_{n}}{{\gamma }_{n}-{\gamma }_{i}},{c}_{\nu }=-\frac{{c}_{i}{\gamma }_{n}}{{\gamma }_{n}-{\gamma }_{i}}$$ and *γ*_*μ*_ = *γ*_*i*_, *γ*_*ν*_ = *γ*_*n*_.
2. If the initial term is a simple exponential and *γ*_*i*_ = *γ*_*n*_, then
  3 $${c}_{i}\theta (t){e}^{-{\gamma }_{i}t}\ast {\gamma }_{n}\theta (t){e}^{-{\gamma }_{n}t}={c}_{\mu }\theta (t)t{e}^{-{\gamma }_{\mu }t},$$
  with *c*_*μ*_ = *c*_*i*_*γ*_*i*_ and *γ*_*μ*_ = *γ*_*i*_.
3. If the initial term is a ${c}_{i}\theta (t){t}^{n}{e}^{-{\gamma }_{i}t}$ term and *γ*_*i*_ = *γ*_*μ*_, then
  4 $${c}_{i}\theta (t){t}^{n}{e}^{-{\gamma }_{i}t}\ast {\gamma }_{n}\theta (t){e}^{-{\gamma }_{n}t}={c}_{\mu }\theta (t){t}^{n+1}{e}^{-{\gamma }_{\mu }t},$$
  with $${c}_{\mu }=\frac{{c}_{i}{\gamma }_{i}}{n+1}$$ and *γ*_*μ*_ = *γ*_*i*_.
4. If the initial term is a ${c}_{i}\theta (t){t}^{n}{e}^{-{\gamma }_{i}t}$ term and *γ*_*i*_ ≠ *γ*_*μ*_, then
  5 $${c}_{i}\theta (t){t}^{n}{e}^{-{\gamma }_{i}t}\ast {\gamma }_{n}\theta (t){e}^{-{\gamma }_{n}t}={c}_{\mu }\theta (t){t}^{n}{e}^{-{\gamma }_{\mu }t}+{c}_{\nu }(\theta (t){t}^{n-1}{e}^{-{\gamma }_{i}t}\ast \theta (t){e}^{-{\gamma }_{n}t}),$$
  where $${c}_{\mu }=\frac{{c}_{i}{\gamma }_{n}}{{\gamma }_{n}-{\gamma }_{i}},{\gamma }_{\mu }={\gamma }_{i}$$, and $${c}_{\nu }=-n\frac{{c}_{i}{\gamma }_{n}}{{\gamma }_{n}-{\gamma }_{i}}$$.

Additional terms in the sum in equation (1) can be introduced by keeping track of the index *m* of the summation for every term and selectively convolving new exponentials only with the corresponding terms.

### Kernel-based simulations of activity

Using the kernels fitted from our functional data, we can simulate neural activity without making any further assumptions about the dynamical equations of the network of neurons. To compute the response of a neuron *i* to the stimulation of a neuron *j*, we simply convolve the kernel *k*_*i*,*j*_(*t*) with the activity Δ*F*_*j*_(*t*) induced by the stimulation in neuron *j*. The activity of the stimulated neuron can be either the experimentally observed activity or an arbitrarily shaped activity introduced for the purposes of simulation.

To compute kernel-derived neural activity correlations (Fig. 6), we completed the following steps. (1) We computed the responses of all the neurons *i* to the stimulation of a neuron *j* chosen to drive activity in the network. To compute the responses, for each pair *i*, *j*, we used the kernel ${\langle {k}_{i,j}(t)\rangle }_{{\rm{trials}}}$ averaged over multiple trials. For kernel-based analysis, pairs with connections of *q* > 0.05 were considered not connected. We set the activity Δ*F*_*j*_(*t*) in the driving neuron to mimic an empirically observed representative activity transient. (2) We computed the correlation coefficient of the resulting activities. (3) We repeated steps 1 and 2 for a set of driving neurons (all or top-n neurons, as in Fig. 6). (4) For each pair *k*, *l*, we took the average of the correlations obtained by driving the set of neurons *j* in step 3.

### Anatomy-derived simulations of activity

Anatomy-derived simulations were performed as described previously^47^. In brief, this simulation approach uses differential equations to model signal transmission through electrical and chemical synapses and includes a nonlinear equation for synaptic activation variables. We injected current in silico into individual neurons and simulated the responses of all the other neurons. Anatomy-derived responses (Fig. 3) of the connection from neuron *j* to neuron *i* were computed as the peak of the response of neuron *i* to the stimulation of *j*. Anatomy-based predictions of spontaneous correlations in Fig. 6 were calculated analogously to kernel-based predictions.

In one analysis in Fig. 3d, the synapse weights and polarities were allowed to float and were fitted from the functional measurements. In all other cases, synapse weights were taken as the scaled average of three adult connectomes^1,6^ and an L4 connectome^6^, and polarities were assigned on the basis of a gene-expression analysis of ligand-gated ionotropic synaptic connections that considered glutamate, acetylcholine and GABA neurotransmitter and receptor expression, as performed in a previous study^37^ and taken from CeNGEN^38^ and other sources. Specifically, we used a previously published dataset (S1 data in ref. ^37^) and aggregated polarities across all members of a cellular subtype (for example, polarities from source AVAL and AVAR were combined). In cases of ambiguous polarities, connections were assumed to be excitatory, as in the previous study^37^. For other biophysical parameters we chose values commonly used in *C. elegans* modelling efforts^9,30,47,73^.

### Characterizing stereotypy of functional connections

To characterize the stereotypy of a neuron pair’s functional connection, its kernels were inspected. A kernel was calculated for every stimulus-response event in which both the upstream and downstream neuron exhibited activity that exceeded a threshold. At least two stimulus-response events that exceeded this threshold were required to calculate their stereotypy. The general strategy for calculating stereotypy was to convolve different kernels with the same stimulus inputs and compare the resulting outputs. The similarity of two outputs is reported as a Pearson’s correlation coefficient. Kernels corresponding to different stimulus-response events of the same pair of neurons were compared with one another round-robin style, one round-robin each for a given input stimulus. For inputs we chose the set of all stimuli delivered to the upstream neuron. The neuron-pairs stereotypy is reported as the average Pearson’s correlation coefficient across all round-robin kernel pairings and across all stimuli.

### Rise time of kernels

The rise time of kernels, shown in Fig. 5c and Extended Data Fig. 6d, was defined as the interval between the earliest time at which the value of the kernel was 1/*e* its peak value and the time of its peak (whether positive or negative). The rise time was zero if the peak of the kernel was at time *t* = 0. However, saturation of the signal transmission can make kernels appear slower than the connection actually is. For example, the simplest instantaneous connection would be represented by a single decaying exponential in equation (1), which would have its peak at time *t* = 0. However, if that connection is saturating, a second, opposite-sign term in the sum is needed to fit the kernel. This second term would make the kernel have a later peak, thereby masking the instantaneous nature of the connection. To account for this effect of saturation, we removed terms representing saturation from the kernels and found the rise time of these ‘non-saturating’ kernels.

### Screen for purely extrasynaptic-dependent connections

To find candidate purely extrasynaptic-dependent connections, we considered the pairs of neurons that are connected in WT animals (*q*^WT^ < 0.05) and non-connected in *unc-31* animals (${q}_{{\rm{eq}}}^{{\rm{unc-31}}}$ < 0.05, with the additional condition *q*^unc−31^ > 0.05 to exclude very small responses that are nonetheless significantly different from the control distribution). We list these connections and provide additional examples in Extended Data Fig. 9.

Using a recent neuropeptide–GPCR interaction screen in *C. elegans*^52^ and gene-expression data from CeNGEN^38^, we find putative combinations of neuropeptides and GPCRs that can mediate those connections (Supplementary Table 1). We produced such a list of neuropeptide and GPCR combinations using the Python package Worm Neuro Atlas (https://github.com/francescorandi/wormneuroatlas). In the list, we only include transcripts from CeNGEN detected with the highest confidence (threshold 4), as described previously^51^. For each neuron pair, we first searched the CeNGEN database for neuropeptides expressed in the upstream neuron, then identified potential GPCR targets for each neuropeptide using information from previous reports^52,74^, and finally went back to the CeNGEN database to find whether the downstream neuron in the pair was among the neurons expressing the specific GPCRs. The existence of potential combinations of neuropeptide and GPCR putatively mediating signalling supports our observation that communication in the candidate neuron pairs that we identify can indeed be mediated extrasynaptically through neuropeptidergic machinery.

### Reporting summary

Further information on research design is available in the Nature Portfolio Reporting Summary linked to this article.

## Online content

Any methods, additional references, Nature Portfolio reporting summaries, source data, extended data, supplementary information, acknowledgements, peer review information; details of author contributions and competing interests; and statements of data and code availability are available at 10.1038/s41586-023-06683-4.

### Supplementary information

Supplementary Information Supplementary Background, Validation, Discussion and References.

Reporting Summary

Supplementary Table 1 Lists of neuropeptide and GPCR combinations for putative purely extrasynaptic-dependent signalling pairs of neurons. Neuropeptide and GPCR combinations were automatically generated based on data from^38,52^, following (Ripoll-Sanchez et al., 2022).

Supplementary Video 1 M1 stimulation evokes pharyngeal muscle contraction. Videos from four animals are shown during signal propagation measurements. In each example, neuron M1 is optogenetically stimulated for 0.5 s and pharyngeal muscle contractions are visible by their effect on the location of neighbouring neurons. M1 is known to release acetylcholine via chemical synapse onto pharyngeal muscles (Franks et al., 2009, Sando et al., 2021). Top panel shows z-projection of tagRFP-T. Bottom panel shows z-projection of GCaMP6 fluorescence. Cross-hairs indicate neuron currently targeted for stimulation.

### Source data

Source Data Fig. 2

Source Data Fig. 3

Source Data Fig. 4

Source Data Fig. 5

Source Data Fig. 6

Source Data Extended Data Fig. 2

**Extended Data Fig. 3:**
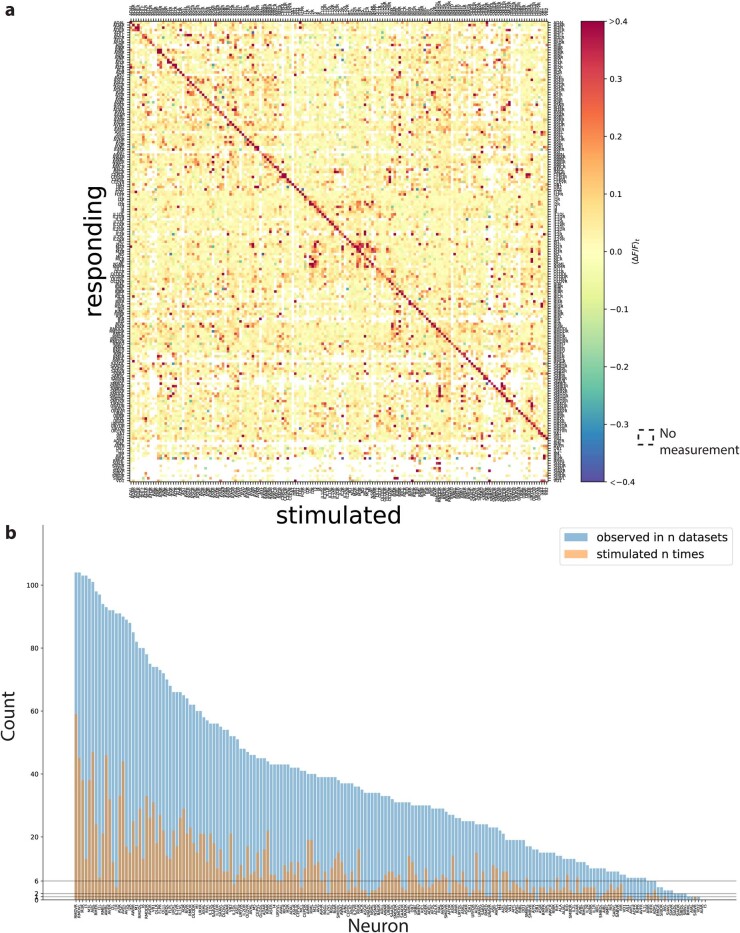
Signal propagation map. **a**, Mean amplitude of neural activity in a post-stimulus time window (${\langle \Delta F/{F}_{0}\rangle }_{t}$) averaged across trials and individuals for WT background. White indicates no measurement. (*n* = 113 animals) For a measurement to be included, a stimulus event is required to evoke a response in the stimulated neuron. Same measurements as in Fig. 2a, but here all neurons that respond are shown, even if they are never stimulated. **b**, Number of stimulation events (orange) and number of datasets (animals) in which the neuron was observed (blue) for each neuron is shown. Source Data

Source Data Extended Data Fig. 3

**Extended Data Fig. 4:**
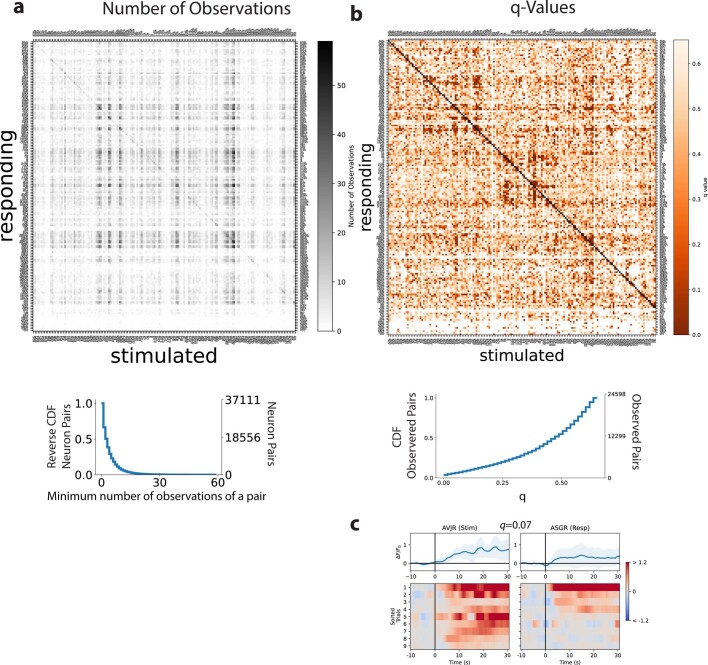
Observations and false discovery rate of neuron pairs in the signal propagation map. **a**, Number of observations made of each neuron pair for WT-background animals. To be considered an observation, the upstream neuron must have been stimulated, calcium imaging of both the upstream and downstream neuron must have been recorded, both neurons must have been unambiguosly identified and the upstream neuron must have exhibited an autoresponse. Sorted as in Extended Data Fig. 3a. Reverse cumulative distribution is also shown (bottom) and reports the fraction of pairs (number of observed pairs divided by the total number of possible pairs of neurons in the head). **b**, *q* values are shown for each neuron pair. *q* values report the false discovery rate of finding a functional connection. They provide a metric of significance for assessing whether a neuron pair is functionally connected based on the number of observations and the magnitude of the response transients, taking into consideration the number of multiple hypotheses tested. Cumulative distribution is also shown (bottom). **c**, ASGR often exhibits activity immediately following stimulation of AVJR, but because its *q* value is greater than 0.05, it does not meet the stringent statistical threshold to be deemed “functionally connected”. Top: mean (blue) and s.d. (shading) across trials and animals. Source Data

Source Data Extended Data Fig. 4

**Extended Data Fig. 5:**
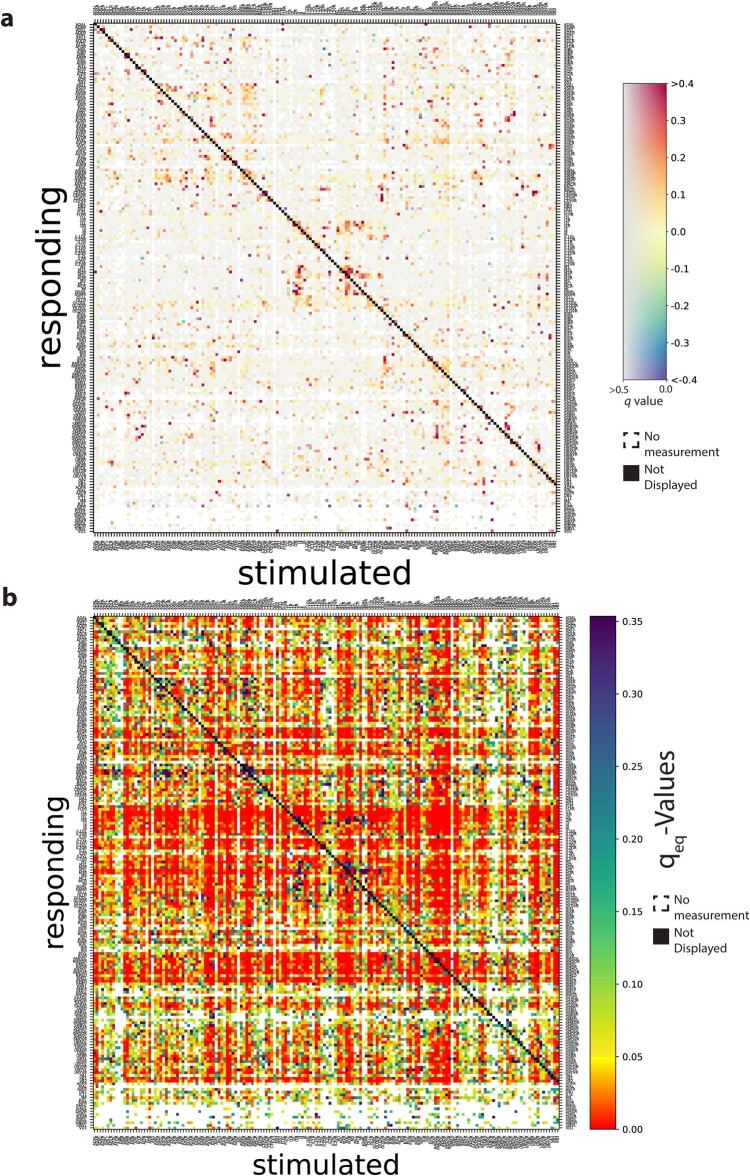
Signal propagation map showing false discovery rates for functional connections and non-connections. **a**, Map of functional connections showing downstream calcium response amplitude and false discovery rate for WT. Same as Fig. 2 except here neurons that are observed but not stimulated are also included. Note the colour bar has two axes. Mean amplitude of neural activity in a post-stimulus time window (${\langle \Delta F/{F}_{0}\rangle }_{t}$) averaged across trials and individuals is shown. *q* value reports false discovery rate (more grey is less significant). White indicates no measurement. Autoresponse is required for inclusion and not displayed (black diagonal). (*n* =113 animals). **b**, Map of functionally not connected pairs. The false discovery rate, *q*_eq_, is reported for declaring a neuron pair to be *not* functionally connected. Lower *q*_eq_ (more red) indicates higher confidence that the observed downstream calcium activity is equivalent within a bound *ϵ* to a null distribution of spontaneous activity. The false discovery rate takes into consideration the amplitude of the calcium transient, the number of observations and the number of hypotheses tested. Source Data

Source Data Extended Data Fig. 5

**Extended Data Fig. 6:**
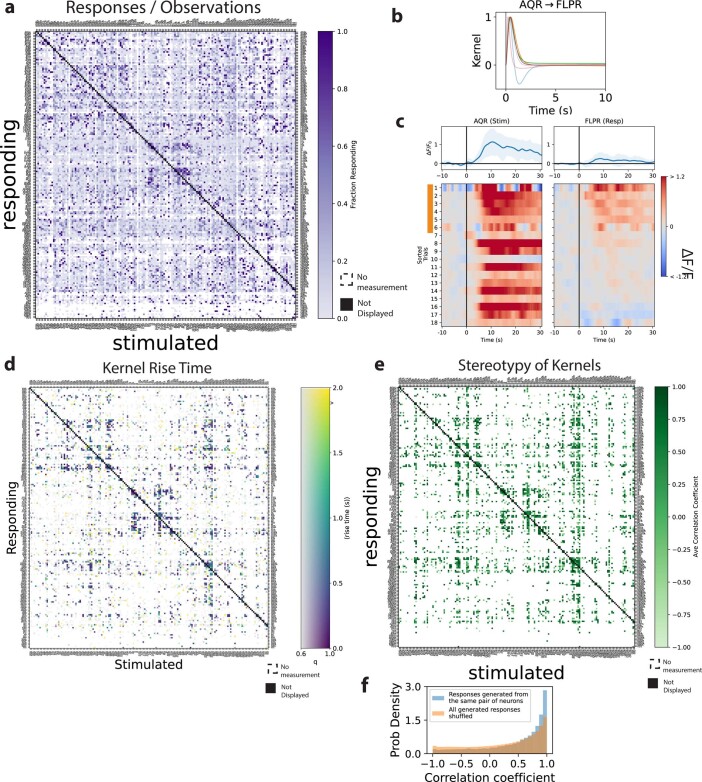
Timescales and variability of measured functional connectivity. WT. **a**, The fraction of stimulation events that evoked a downstream “response” is shown for each neuron pair. To be classified as a “response” requires a sufficiently large calcium transient amplitude and derivative. Autoresponses are required and not shown (black diagonal). **b**, Kernels are functions that return the downstream neuron’s activity when convolved with the upstream neuron’s activity. Kernels capture properties of the connection independent of variability in the upstream neuron’s autoresponse. **c**, Kernels are shown for each FLP response to AQR stimulation. Kernels are only calculated for stimuli that evoked downstream ‘responses’ (indicated in orange). Top: mean (blue) and s.d. (shading) across trials and animals. **d**, Kernel rise time for each measured neuron pair in WT is a metric of signal propagation speed. **e**, The stereotypy of kernels within each neuron pair is reported by calculating the average correlation coefficient among them. Only neuron pairs with at least two kernels are considered. **f**, Distribution of the correlation-coefficients of convolved kernels, within each pair of neurons (blue, n = 30,406), and across all kernels measured regardless of neuron pair (orange, *n* = 113,880,912). Source Data

Source Data Extended Data Fig. 6

**Extended Data Fig. 7:**
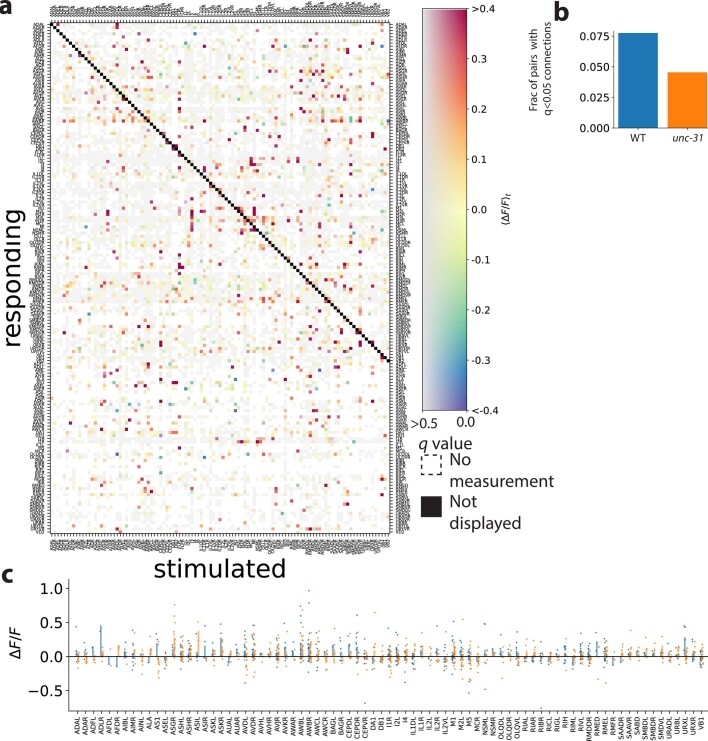
Signal propagation of the *unc-31* background, with defects in dense-core-vesicle-mediated extrasynaptic signalling. **a**, Same format as Extended Data Fig. 5a. Mean amplitude of neural activity in a post-stimulus time window (${\langle \Delta F/{F}_{0}\rangle }_{t}$) averaged across trials and individuals is shown. *q* value reports false discovery rate and is a metric of significance (more grey is less significant). White indicates no measurement. Autoresponse is required and not displayed (black diagonal). (*n* = 18 animals). **b**, *unc-31* mutants had a smaller proportion of measured pairwise neurons that were functionally connected (*q* < 0.05) than WT (considering only those pairs for which data is present in both WT and *unc-31* mutants). **c**, Responses to RID stimulation are shown for WT (blue) and *unc-31* (orange). Points are responses, bar is mean across trials and animals. Neurons with the smallest amplitude responses are not shown. Corresponding traces for ADLR, AWBR and URXL are shown in Fig. 4. As in that figure, responses here are shown even for those cases when RID’s calcium activity was not measured and therefore do not appear in **a**. Different inclusion criteria are used here to accommodate cases in which the tagRFP-T expression is dim, as described in the Methods. Source Data

**Extended Data Fig. 8:**
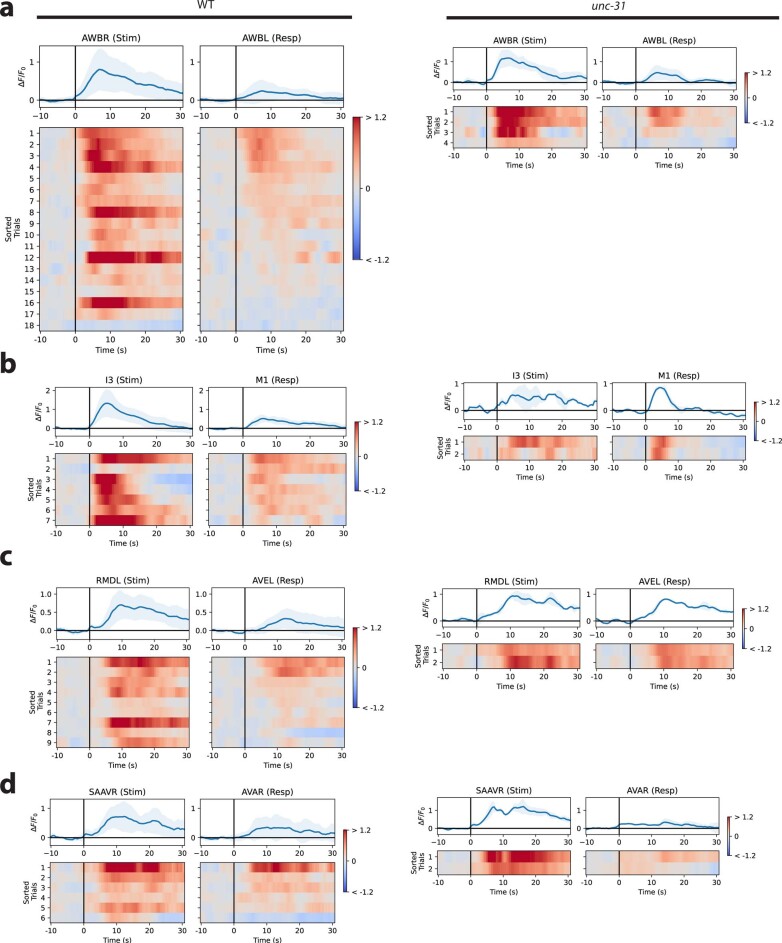
Neural responses for some pairs are similar in WT and *unc-31*-mutant animals. Paired stimulus and response traces of selected neuron pairs with monosynaptic gap junctions (**a**–**c**) or monosynaptic chemical synapses (**d**) are shown in a WT background (left) and in a *unc-31*-mutant background (right). Top: mean (blue) and s.d. (shading) across trials and animals.

Source Data Extended Data Fig. 7

**Extended Data Fig. 9:**
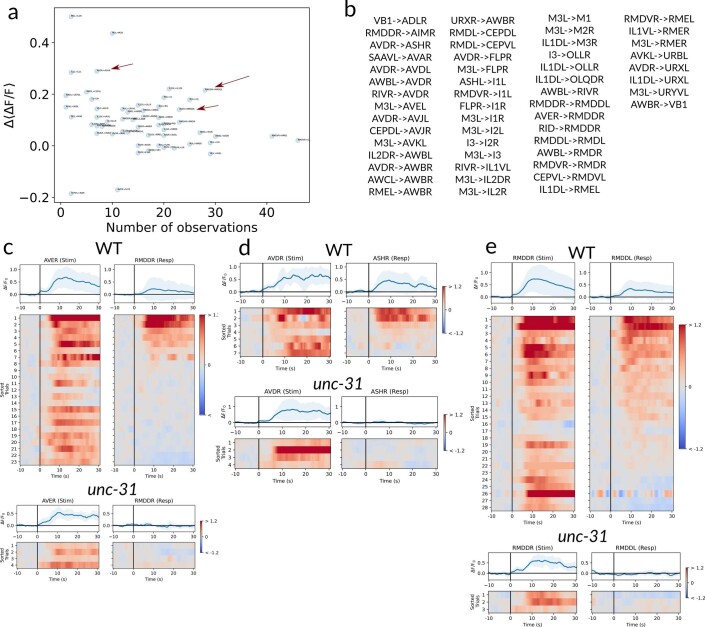
Examples of candidate purely extrasynaptic pairs. **a**, Change in activity Δ⟨Δ*F*/*F*⟩ versus number of WT observations for our candidate purely extrasynaptic-dependent pairs. Arrows indicate examples shown below. **b**, List of candidate entirely extrasynaptic-dependent connections. Relevant neuropeptide GPCR expression is listed in Supplementary Table 1, compiled from refs. ^38,52^, following ref. ^51^. **c**–**e**, Paired responses in WT and *unc-31* animals for the candidate extrasynaptic pairs AVER–RMDDR (**c**), AVDR–ASHR (**d**) and RMDDR–RMDDL (**e**), selected among all the candidates as illustrated in **a**. Top: average (blue) and s.d. (shading) across trials and animals. Source Data

Source Data Extended Data Fig. 9

## Figures and Tables

**Extended Data Table 1:**
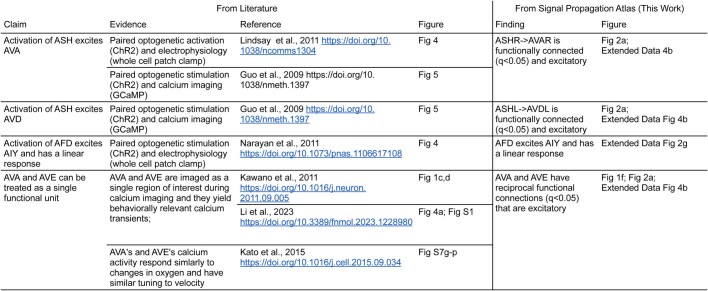
Selected instances of agreement between measured signal propagation and previously reported functional measurements

Comparisons between selected findings from the literature and the current work.

## Data Availability

Machine-readable datasets containing the measurements from this work are publicly accessible through on Open Science Foundation repository at 10.17605/OSF.IO/E2SYT. Interactive browsable versions of the same data are available online at https://funconn.princeton.edu and http://funsim.princeton.edu. CeNGeN data^38^ were accessed through http://www.cengen.org/cengenapp/. Source data are provided with this paper.

## Code availability

All analysis code is publicly available at https://github.com/leiferlab/pumpprobe (10.5281/zenodo.8312985), https://github.com/leiferlab/wormdatamodel (10.5281/zenodo.8247252), https://github.com/leiferlab/wormneuronsegmentation-c (10.5281/zenodo.8247242) and https://github.com/leiferlab/wormbrain (10.5281/zenodo.8247254). Hardware acquisition code is available at https://github.com/leiferlab/pump-probe-acquisition (10.5281/zenodo.8247258).
