# Supplementary material for: Neural signal propagation atlas of Caenorhabditis elegans
Source: Nature. 2023 Nov 1;623(7986):406–14. doi: 10.1038/s41586-023-06683-4 (PMC10632145; doi:10.1038/s41586-023-06683-4)
Supplement: Supplementary file 1 — Supplementary Background, Validation, Discussion and References. [file 41586_2023_6683_MOESM1_ESM.pdf]

nature portfolio

https://doi.org/10.1038/s41586-023-06683-4

## Supplementary information

# Neural signal propagation atlas of *Caenorhabditis elegans*

In the format provided by the authors and unedited

nature portfolio

https://doi.org/10.1038/s41586-023-06683-4

# Supplementary information

# Neural signal propagation atlas of *Caenorhabditis elegans*

In the format provided by the authors and unedited

# Supplementary Text: Neural Signal Propagation atlas of C. elegans

Francesco Randi, Anuj K Sharma, Sophie Dvali and Andrew M Leifer

Corresponding author: leifer@princeton.edu

## Contents

0.1 Supplementary Background . . . . . . . . . . i

Other names for signal propagation measurements • Extrasynaptic signaling background

0.2 Supplementary Validation . . . . . . . . . . ii

Spatial Validation of Single Neuron Targeting • Temporal Validation of Single Neuron Targeting

0.3 Interpretation of Screen for Extrasynaptic Signaling . . . . . . . . . . iii

0.4 Supplementary Discussion . . . . . . . . . . iv

Potential role in validating predictions from gene expression

0.5 References for Supplementary Text . . . . . . . . . . iv

## 0.1 Supplementary Background

### *0.1.1 Other names for signal propagation measurements*

Previous efforts to measure signal propagation via perturbations have been variously called, “influence mapping” [75], “projective fields” [76], “evoked brain connectivity” [77] and “functional connectivity” [22]. These are not to be confused with approaches that measure correlations in neural activity— also often referred to as “functional connectivity.”

### *0.1.2 Extrasynaptic signaling background*

Extrasynaptic signaling, sometimes called “volume transmission” [78], occurs either because the transmitter is released far from the cleft, for example via dense core vesicle, or because transmitter released at the cleft spills out into the milieu. Extrasynaptic signaling has been reported for GABA [79, 80], NMDA [81], acetylcholine [82], monoamines [49], and neuropeptides [83]. In all cases, extrasynaptic signaling is not visible from anatomy and therefore forms additional wireless layers of communication in the nervous system [49]. We are motivated to investigate extrasyaptic signaling of neuropeptides in particular because gene expression of neuropeptides and neuropeptide receptors are ubiquitous across not only the *C. elegans* nervous system [38, 51, 52] but also in mammalian cortex [50]. Neuropeptides are typically released via dense core vesicles [84] and are not required to be released at the synaptic cleft. Here we refer to dense-core-vesicle mediated signaling as extrasynaptic because it is commonly observed far from the synapse, however we cannot exclude the possibility that dense-core vesicles may also be released at the synapse. In this work we study mutants defective for UNC-31/CAPS, a protein involved in dense core vesicle release. In *C. elegans*, defects in UNC-31/CAPS are not expected to disrupt chemical or electrical synapses [53].

Extrasynaptic signaling is often associated with neuromodulation and is assumed to alter excitability or modulate synaptic properties that change how neurons respond to input [16, 83] often over long timescales, though not always [85]. But

extrasynpatic transmission can also directly evoke activity in downstream neurons. Our signal propagation measurements are not designed to directly detect changes to a neuron's excitability and instead such changes might appear as variability in our measured responses. But our measurements should detect instances where extrasynaptic signaling evokes activity, and that is what we observe, Fig. 5b.

## 0.2 Supplementary Validation

### *Validation of GUR-3/PRDX-2*

We chose to use the GUR-3/PRDX-2 purple-light activatable optogenetic system [24, 25] in combination with GCaMP6s [26] in order avoid optical crosstalk challenges that are common in other pairs of indicators and actuators [19, 20, 86, 87, 88]. By using the GUR-3/PRDX-2 actuator, we are able to image GCaMP6s at 505 nm [26] without detectable activation [23]. GUR-3/PRDX-2 is a gustatory receptor homolog endogenous to *C. elegans* and is thought to respond to reactive oxygen species generated by light. Several lines of evidence provide reassurances that light activation of GUR-3/PRDX-2 evokes neural signaling as expected. For example, light-activation of GUR-3/PRDX-2 in I2 was shown to inhibit pharyngeal pumping by release of glutamate [89]. In control experiments with freely moving animals, we observe that light activation of GUR-3/PRDX-2 expressed in AVA evokes reversals (Extended Data Fig. 2h, methods), as expected [45, 60]. Furthermore, activation of cholinergic motor neurons M1 during the course of our signal propagation measurements evoked pharyngeal muscle contraction (Supplementary Video 1). Since M1 is known to release acetylcholine via chemical synapse onto pharyngeal muscles [90, 91] this is evidence that the stimulation condition we use in our experiments are well suited to evoke typical synaptic release of classical neurotransmitters.

### *0.2.1 Spatial Validation of Single Neuron Targeting*

To activate single neurons without also activating their neighbors, we spatially restricted the optogenetic excitation volume in three dimensions to the typical size of a *C. elegans* cell soma using temporal focusing [92, 19]. We validated our targeting several ways. We characterized the point-spread function of our spatially restricted 2 photon stimulation spot using fluorescent beads and showed that it matched our desired size in all three dimensions (Extended Data Fig. 2a). We performed photobleaching experiments (at reduced power) and showed that only targeted neurons were appreciably photobleached, regardless of whether we targeted neurons near (Extended Data Fig. 2c) or far from the objective (Extended Data Fig. 2d). We demonstrated in vivo that stimulation inside the cell soma resulted in evoked calcium transients, but immediately outside the soma (4 microns away) resulted in no neural activation (Extended Data Fig. 2e).

And notably, an analysis of our signal propagation atlas measurements is also consistent with spatially restricted stimulation. Stimulation events in the course of our signal propagation measurements often evoked calcium responses in the targeted neuron, but for neurons in the immediate vicinity of the targeted neuron, ∼75% of stimulation events did not show obvious activation (Extended Data Fig. 2i,j), which places a worst-case-scenario extreme upper bound on off-target stimulation at ∼25%. In practice, we suspect most of these nearby responses are not the result of off-target stimulation but rather are legitimate downstream responses that result from the spatially restricted on-target activation of the targeted neuron.

#### *0.2.2 Temporal Validation of Single Neuron Targeting*

By inspecting the timing of stimulation artifacts in our calcium traces, such as those visible in Extended Data Fig. 2e, we concluded that there is occasionally a temporal jitter of up to one imaging volume (corresponding to 0.5 s) between the time of the stimulation and the time we report the stimulation to have occurred. This means there can be an up to 1 volume jitter in the temporal alignment of stimulation to calcium activity in figures such as Fig. 1e and their average response. This has no impact on measurements of signal propagation time scales (Fig. 5b) or on calculations of the kernels (Extended Data Fig. 6b,d,e-f) because those are all calculated by comparing only the calcium activity of the upstream and downstream neurons, without regard to when the stimulus was applied.

### 0.3 Interpretation of Screen for Extrasynaptic Signaling

The screen for purely extrasynatic signaling identifies only those subset of neuron pairs that pass our statistical threshold to be deemed functionally connected in WT ($q < 0.05$) and also our threshold to be functionally non-connected in *unc-31* ($q_{\text{eq}} < 0.05$)). Additional neuron pairs likely signal extrasynaptically but don't meet this stringent thresholds because we lack sufficient observations. Other pairs likely signal through multiple parallel paths including both synaptic and extrasynaptic ones, or exhibit co-transmission of both, but these would not necessarily be detected by the screen, which effectively requires all signaling paths to cease in *unc-31*.

Given the degree of anatomical connectedness (any neuron is anatomically connected to any other in no more than four hops) it is striking that we found so many neuron pairs that pass our stringent test for extrasynaptic dependence across all paths. We note that pairs that pass our screen could include signaling paths that involve synaptic signaling *in series* with extrasynaptic signaling, as this would still be purely extrasynaptic dependent. Our screen assumes that the *unc-31* mutant is defective only for dense core vesicle release, as reported [53]. *unc-31*-dependent defects in any additional signaling modalities, or *unc-31*-dependent changes to wiring, would present a confound to interpreting these measurements.

We interpret passing our screen's criteria (functional connection in WT and a non-functional connection in *unc-31*) as evidence of acute extrasynaptic signaling on short timescales (30s or less). An alternative explanation is that instead neuromodulators regulate neural excitability (possibly on long timescales) in an *unc-31*-dependent manner and we are merely observing the presence and absence of this modulation on synaptic signaling. This explanation is not supported by the evidence. The decrease in signaling in *unc-31* cannot be explained by changes to the upstream neuron's excitability because the upstream neuron's autoresponse is measured and required to be present. Similarly, our measurements are inconsistent with a dramatic decrease in excitability of the downstream neuron in *unc-31* because for the vast majority (85%) of the neuron pairs that pass our screen, the downstream neuron still responds to synaptic inputs from at least one other neuron in *unc-31*. For example, AVDR->AVDL appears in our screen (Fig. 5d) yet AVDL still responds to activation of other neurons such as AVJL ($q < 0.05$ in both WT and *unc-31*, Fig. 2a, Extended Data Fig. 7a), which is inconsistent with a dramatic decrease in AVDL excitability. These observations are consistent with our interpretation that the lack of a response results from an *unc-31*-dependent loss of acute short-timescale extrasynaptic signaling.

We note that we cannot rule out an alternative interpretation in which *unc-31*-dependent neuromodulators specifically upregulates the function of some of the synapses onto a downstream neuron but not others and does not alter the upstream or downstream neuron's overall excitability. Under this interpretation we may be observing wired synaptic signaling that is no longer upregulated in the *unc-31* animal. Although we cannot rule it out, we find this interpretation less compelling because: 1) it would require *unc-31*-dependent regulation of an individual synapse to a downstream neuron without also affecting other synapses onto it, or their overall excitability, and we are not familiar with molecular mechanisms for this, although they may exist, 2) many of the neuron pairs that pass the screen have no direct wired connections suggesting that at best synaptic signaling is indirect, and 3) many of the neuron pairs that pass the screen do express appropriate peptide receptor pairs that are consistent with extrasynaptic signaling.

### 0.4 Supplementary Discussion

Our signal propagation map provides a lower bound on the number of functional connections. For example, our analysis has fewer observations of neurons whose tagRFP-T expression is consistently too dim or whose color and location pattern is too difficult to identify, and so we are less likely to have the statistical power to declare those neurons functionally connected. We also are limited to calcium activity in the nucleus, and therefore omit compartmentalized calcium dynamics [93]. And we see only neural responses that result in calcium transients. For example, while we see instances of inhibition, we suspect we see only those instances in which a tonically active neuron is inhibited.

The neural dynamics we observe are slow but no slower than typical calcium responses to natural stimuli such as odor delivery [94]. The slow dynamics likely result from the slow graded potentials in *C. elegans* [48, 95], the slower calcium dynamics of the nucleus [35] and the slow rise and fall time of GCaMP6s [26] and GUR-3/PRDX-2 [24]. We identify fast signal transmission, even in slow dynamics, by fitting kernels to relate the dynamics of upstream and downstream neurons.

#### *0.4.1 Potential role in validating predictions from gene expression*

Signal propagation measurements may potentially play an important role in validating predictions of peptidergic signaling from gene express [51, 52, 96]. The expression of appropriate neuropeptide/GPCR gene combinations indicates only that the molecular machinery for extrasynaptic peptidergic signaling is present. By contrast, functional measurements like those performed here provide the more direct evidence that extrasyanaptic signaling actually occurs. Moreover functional measurements resolve whether such signaling directly evokes neural responses, or for example, whether they only modulate neural excitability over longer-timescales.

### 0.5 References for Supplementary Text

## References

**75.** Panzeri, S., Moroni, M., Safaai, H. & Harvey, C. D. The structures and functions of correlations in neural population codes. *Nat. Rev. Neurosci.* 1–17, DOI: 10.1038/s41583-022-00606-4 (2022). Publisher: Nature Publishing Group.

**76.** Lehky, S. R. & Sejnowski, T. J. Network model of shape-from-shading: neural function arises from both receptive and projective fields. *Nature* **333**, 452–454, DOI: 10.1038/333452a0 (1988). Number: 6172 Publisher: Nature Publishing Group.

**77.** Lepage, K. Q., Ching, S. & Kramer, M. A. Inferring evoked brain connectivity through adaptive perturbation. *J. Comput. Neurosci.* **34**, 303–318, DOI: 10.1007/s10827-012-0422-8 (2013).

**78.** Agnati, L. F., Zoli, M., Strömberg, I. & Fuxe, K. Intercellular communication in the brain: wiring versus volume transmission. *Neuroscience* **69**, 711–726, DOI: 10.1016/0306-4522(95)00308-6 (1995).

**79.** Brickley, S. G. & Mody, I. Extrasynaptic GABAA Receptors: Their Function in the CNS and Implications for Disease. *Neuron* **73**, 23–34, DOI: 10.1016/j.neuron.2011.12.012 (2012).

**80.** Shen, Y. *et al.* An extrasynaptic GABAergic signal modulates a pattern of forward movement in Caenorhabditis elegans. *eLife* **5**, e14197, DOI: 10.7554/eLife.14197 (2016). Publisher: eLife Sciences Publications, Ltd.

**81.** Hardingham, G. E. & Bading, H. Synaptic versus extrasynaptic NMDA receptor signalling: implications for neurodegenerative disorders. *Nat. Rev. Neurosci.* **11**, 682–696, DOI: 10.1038/nrn2911 (2010). Number: 10 Publisher: Nature Publishing Group.

**82.** Lu, Y. *et al.* Extrasynaptic signaling enables an asymmetric juvenile motor circuit to produce symmetric undulation. *Curr. Biol.* **32**, 4631–4644.e5, DOI: 10.1016/j.cub.2022.09.002 (2022).

**83.** Taghert, P. H. & Nitabach, M. N. Peptide neuromodulation in invertebrate model systems. *Neuron* **76**, 82–97, DOI: 10.1016/j.neuron.2012.08.035 (2012).

**84.** Pelletier, G., Leclerc, R. & Dupont, A. Electron microscope immunohistochemical localization of substance P in the central nervous system of the rat. *J. Histochem. & Cytochem.* **25**, 1373–1375, DOI: 10.1177/25.12.925343 (1977). Publisher: Journal of Histochemistry & Cytochemistry.

**85.** Golowasch, J. & Marder, E. Proctolin activates an inward current whose voltage dependence is modified by extracellular Ca2+. *J. Neurosci.* **12**, 810–817, DOI: 10.1523/JNEUROSCI.12-03-00810.1992 (1992). Publisher: Society for Neuroscience Section: Articles.

**86.** Yang, W., Carrillo-Reid, L., Bando, Y., Peterka, D. S. & Yuste, R. Simultaneous two-photon imaging and two-photon optogenetics of cortical circuits in three dimensions. *eLife* **7**, DOI: 10.7554/eLife.32671 (2018).

**87.** Zhang, Z., Russell, L. E., Packer, A. M., Gauld, O. M. & Häusser, M. Closed-loop all-optical interrogation of neural circuits in vivo. *Nat. Methods* **15**, 1037, DOI: 10.1038/s41592-018-0183-z (2018).

**88.** Russell, L. E. *et al.* All-optical interrogation of neural circuits in behaving mice. *Nat. Protoc.* 1–42, DOI: 10.1038/s41596-022-00691-w (2022). Publisher: Nature Publishing Group.

**89.** Bhatla, N., Droste, R., Sando, S. R., Huang, A. & Horvitz, H. R. Distinct Neural Circuits Control Rhythm Inhibition and Spitting by the Myogenic Pharynx of C. elegans. *Curr. biology: CB* **25**, 2075–2089, DOI: 10.1016/j.cub.2015.06.052 (2015).

**90.** Franks, C. J., Murray, C., Ogden, D., O'Connor, V. & Holden-Dye, L. A comparison of electrically evoked and channel rhodopsin-evoked postsynaptic potentials in the pharyngeal system of Caenorhabditis elegans. *Invertebr. neuroscience: IN* **9**, 43–56, DOI: 10.1007/s10158-009-0088-8 (2009).

**91.** Sando, S. R., Bhatla, N., Lee, E. L. & Horvitz, H. R. An hourglass circuit motif transforms a motor program via subcellularly localized muscle calcium signaling and contraction. *eLife* **10**, e59341, DOI: 10.7554/eLife.59341 (2021). Publisher: eLife Sciences Publications, Ltd.

**92.** Andrasfalvy, B. K., Zemelman, B. V., Tang, J. & Vaziri, A. Two-photon single-cell optogenetic control of neuronal activity by sculpted light. *Proc. Natl. Acad. Sci.* **107**, 11981–11986, DOI: 10.1073/pnas.1006620107 (2010). Publisher: Proceedings of the National Academy of Sciences.

**93.** Hendricks, M., Ha, H., Maffey, N. & Zhang, Y. Compartmentalized calcium dynamics in a C. elegans interneuron encode head movement. *Nature* **487**, 99–103, DOI: 10.1038/nature11081 (2012).

**94.** Lin, A. *et al.* Functional imaging and quantification of multi-neuronal olfactory responses in C. elegans, DOI: 10.1101/2022.05.27.493772 (2022). Pages: 2022.05.27.493772 Section: New Results.

**95.** Lindsay, T. H., Thiele, T. R. & Lockery, S. R. Optogenetic analysis of synaptic transmission in the central nervous system of the nematode Caenorhabditis elegans. *Nat. Commun.* **2**, 306, DOI: 10.1038/ncomms1304 (2011).

**96.** Taylor, D. L. Chapter 13 Basic Fluorescence Microscopy. *Methods Cell Biol.* **29**, 207–237, DOI: 10.1016/S0091-679X(08)60196-X (1988).

**97.** Li, Z. *et al.* A C. elegans neuron both promotes and suppresses motor behavior to fine tune motor output. *Front. Mol. Neurosci.* **16** (2023).

## Supplementary Material

**Supplementary Spreadsheet 1** Microsoft Excel file lists neuropeptide and GPCR combinations for putative purely extrasynaptic-dependent signaling pairs of neurons. Neuropeptide and GPCR combinations are automatically generated based on data from [52, 38], following [51].

**Supplementary Video 1** M1 stimulation evokes pharyngeal muscle contraction. Video from four animals are shown during signal propagation measurements. In each example, neuron M1 is optogenetically stimulated for 0.5 s and pharyngeal muscle contractions are visible by their effect on the location of neighboring neurons. M1 is known to release acetylcholine via chemical synapse onto pharyngeal muscles [90, 91]. Top panel shows z-projection of tagRFP-T. Bottom panel shows z-projection of GCaMP6 fluorescence. Crosshairs indicate neuron currently targeted for stimulation.
