# Supplementary material for: Neural signal propagation atlas of Caenorhabditis elegans
Source: Nature. 2023 Nov 1;623(7986):406–14. doi: 10.1038/s41586-023-06683-4 (PMC10632145; doi:10.1038/s41586-023-06683-4)
Supplement: Supplementary file 2 — Reporting Summary [file 41586_2023_6683_MOESM2_ESM.pdf]

nature portfolio

Corresponding author(s): Andrew Leifer

Last updated by author(s): 4/10/2023

Reporting Summary

Nature Portfolio wishes to improve the reproducibility of the work that we publish. This form provides structure for consistency and transparency in reporting. For further information on Nature Portfolio policies, see our Editorial Policies and the Editorial Policy Checklist.

Statistics

For all statistical analyses, confirm that the following items are present in the figure legend, table legend, main text, or Methods section.

n/a	Confirmed	
☐	☒	The exact sample size (*n*) for each experimental group/condition, given as a discrete number and unit of measurement
☐	☒	A statement on whether measurements were taken from distinct samples or whether the same sample was measured repeatedly
☐	☒	The statistical test(s) used AND whether they are one- or two-sided<br>*Only common tests should be described solely by name; describe more complex techniques in the Methods section.*
☒	☐	A description of all covariates tested
☐	☒	A description of any assumptions or corrections, such as tests of normality and adjustment for multiple comparisons
☐	☒	A full description of the statistical parameters including central tendency (e.g. means) or other basic estimates (e.g. regression coefficient) AND variation (e.g. standard deviation) or associated estimates of uncertainty (e.g. confidence intervals)
☐	☒	For null hypothesis testing, the test statistic (e.g. *F*, *t*, *r*) with confidence intervals, effect sizes, degrees of freedom and *P* value noted<br>*Give P values as exact values whenever suitable.*
☒	☐	For Bayesian analysis, information on the choice of priors and Markov chain Monte Carlo settings
☒	☐	For hierarchical and complex designs, identification of the appropriate level for tests and full reporting of outcomes
☐	☒	Estimates of effect sizes (e.g. Cohen's *d*, Pearson's *r*), indicating how they were calculated

Our web collection on statistics for biologists contains articles on many of the points above.

Software and code

Policy information about availability of computer code

Data collection	Software to control acquisition hardware is available at https://github.com/leiferlab/pump-probe based
Data analysis	All analysis code is publicly available at https://github.com/leiferlab/pumpprobe (DOI:10.5281/zenodo.8247256), https://github.com/leiferlab/wormdatamodel (DOI:10.5281/zenodo.8247252), https://github.com/leiferlab/wormneuronsegmentation-c (DOI:10.5281/zenodo.8247242), and https://github.com/leiferlab/wormbrain (DOI:10.5281/zenodo.8247254). Hardware acquisition code is available at https://github.com/leiferlab/pump-probe-acquisition (DOI:10.5281/zenodo.8247258).

For manuscripts utilizing custom algorithms or software that are central to the research but not yet described in published literature, software must be made available to editors and reviewers. We strongly encourage code deposition in a community repository (e.g. GitHub). See the Nature Portfolio guidelines for submitting code & software for further information.

Data

Policy information about availability of data

All manuscripts must include a data availability statement. This statement should provide the following information, where applicable:

- Accession codes, unique identifiers, or web links for publicly available datasets
- A description of any restrictions on data availability
- For clinical datasets or third party data, please ensure that the statement adheres to our policy

Machine readable datasets containing the measurements from this work are publicly accessible through on Open Science Foundation repository at https://doi.org/10.17605/OSF.IO/E2SYT . Interactive browseable versions of this same data are available online at https://funconn.princeton.edu and http://funsim.princeton.edu . CeNGeN data was accessed through http://www.cengen.org/cengenapp/.

Human research participants

Policy information about studies involving human research participants and Sex and Gender in Research.

Reporting on sex and gender	N/A
Population characteristics	N/A
Recruitment	N/A
Ethics oversight	N/A

Note that full information on the approval of the study protocol must also be provided in the manuscript.

Field-specific reporting

Please select the one below that is the best fit for your research. If you are not sure, read the appropriate sections before making your selection.

☒ Life sciences ☐ Behavioural & social sciences ☐ Ecological, evolutionary & environmental sciences

For a reference copy of the document with all sections, see nature.com/documents/nr-reporting-summary-flat.pdf

Life sciences study design

All studies must disclose on these points even when the disclosure is negative.

Sample size

No sample-size calculation was performed. We recorded from >113 individual WT-background animals and performed over 20,000 pairwise stimulus response measurements. Sample size was chosen to be many fold larger than typical C. elegans calcium imaging experiments in the field, e.g. Hallinen et al., elife 2021.

Data exclusions

Inclusion and exclusion criteria are described in the "Inclusion criteria" subsection of the Methods, and pasted here:
Stimulation events were included for further analysis if they evoked a detectable calcium response in the stimulated neuron (autoresponse). A classifier determined whether the response was detected by inspecting whether the amplitude of both the DF/F transient and its second derivative exceeded a pair of thresholds. The same threshold values were applied to every animal, strain, neuron and stimulation event, and were originally set to match human perception of a response above noise. Stimulation events that did not meet both thresholds for a contiguous 4 seconds were excluded. RID responses shown in Fig. 4 and Extended Data Fig. 7c are an exception to this policy. RID is visible based on its CyOFP expression, but its tagRFP-T expression is too dim to consistently extract calcium signals. Therefore in Fig. 4 and Extended Data Fig. 7c (but not in other figures, like Fig. 2) responses to RID stimulation were included even in cases where it was not possible to extract a calcium-activity trace in RID.

Neuron traces were excluded from analysis if a human was unable to assign an identity or if the imaging time points were absent in a contiguous segment longer than 5% of the response window due to imaging artifacts or tracking errors. A different policy applies to dim neurons of interest that are not automatically detected by the ``pseudo''-segmentation algorithm in the 3D image used as reference for the pointset registration algorithm. In those cases, we manually added the position of those neurons to the reference 3D image. If these ``added'' neurons are automatically detected in most of the other 3D images, then a calcium activity trace can be successfully produced by the DSMM nonrigid registration algorithm and is treated as any other trace. However, if the ``added'' neurons are too dim to be detected also in the other 3D images and the calcium activity trace cannot be formed for more than 50% of the total time points, the activity trace for those neurons is extracted from the neuron's position as determined from the position of neighboring neurons. In the analysis code, we refer to these as ``matchless'' traces, because the reference neuron is not matched to any detected neuron in the specific 3D image, but its position is just transformed according to the DSMM nonrigid deformation field. In this way, we are able to recover the calcium activity also of some neurons whose tag-RFP-T expression is otherwise too dim to be reliably detected by the ``pseudo''-segmentation algorithm. Responses to RID stimulation shown in Fig. 4 and Extended Data Fig. 7c are an exception to this policy. There, the activity of any neuron for which there is not a trace for more than 50% of the time points is substituted with the corresponding ``matchless'' trace, and not just for the manually added neurons. This is important to be able to show responses of neurons like ADL, which have dim tagRFP-T expression. In the RID-specific case, in

order to exclude responses that become very large solely because of numerical issues in the division by the baseline activity due to the dim tagRFP-T, we additionally introduce a threshold excluding DF/F>2.

Kernels were computed only for stimulation-response events for which the automatic classifier detected responses in both the stimulated and downstream neurons. If the downstream neuron did not show a response, we considered the downstream response to be below the noise level and the kernel to be zero.

Replication

The number of replications for each WT measurement is presented in Supplementary Figure S5a, and additional related information is presented in Supplementary Figure S6.

Randomization

Randomization was not relevant to our study because we are not testing an intervention on individuals, but instead mapping out signal propagation in WT and mutant animals.

Blinding

Humans were blinded to calcium activity when they assigned neurons their identities. An exception is neuron AIY in experiments associated with Supplementary Fig S11. Because AIY's identity is sometimes ambiguous based on its position and color, calcium activity was occasionally used to confirm AIY's identity.

Reporting for specific materials, systems and methods

We require information from authors about some types of materials, experimental systems and methods used in many studies. Here, indicate whether each material, system or method listed is relevant to your study. If you are not sure if a list item applies to your research, read the appropriate section before selecting a response.

Materials & experimental systems

n/a	Involved in the study	
☒	☐	Antibodies
☒	☐	Eukaryotic cell lines
☒	☐	Palaeontology and archaeology
☐	☒	Animals and other organisms
☒	☐	Clinical data
☒	☐	Dual use research of concern

Methods

n/a	Involved in the study	
☒	☐	ChIP-seq
☒	☐	Flow cytometry
☒	☐	MRI-based neuroimaging

Animals and other research organisms

Policy information about studies involving animals; ARRIVE guidelines recommended for reporting animal research, and Sex and Gender in Research

Laboratory animals

C .elegans. Strains used include AML462 and AML508 as described in the "Strains" section of the Materials and Methods.

Wild animals

Only laboratory strains were used.

Reporting on sex

Hermaphrodites were studied because >99.8% of naturally occurring C. elegans are hermaphrodites (Corsi, et al., WormBook 2015)

Field-collected samples

N/A

Ethics oversight

No ethical approval or guidance was required because C. elegans are microscopic invertebrate worms.

Note that full information on the approval of the study protocol must also be provided in the manuscript.
